# Directly Isolated Allogeneic Virus–Specific T Cells in Progressive Multifocal Leukoencephalopathy

**DOI:** 10.1001/jamaneurol.2024.3324

**Published:** 2024-10-07

**Authors:** Nora Möhn, Lea Grote-Levi, Mike P. Wattjes, Agnes Bonifacius, Dennis Holzwart, Franziska Hopfner, Sandra Nay, Sabine Tischer-Zimmermann, Mieke Luise Saßmann, Philipp Schwenkenbecher, Kurt-Wolfram Sühs, Nima Mahmoudi, Clemens Warnke, Julian Zimmermann, David Hagin, Lilia Goudeva, Rainer Blasczyk, Armin Koch, Britta Maecker-Kolhoff, Britta Eiz-Vesper, Günter Höglinger, Thomas Skripuletz

**Affiliations:** 1 Department of Neurology, Hannover Medical School, Hannover, Germany; 2 Department of Diagnostic and Interventional Neuroradiology, Hannover Medical School, Hannover, Germany; 3 Department of Neuroradiology, Charité - Universitätsmedizin Berlin, Corporate Member of Freie Universität Berlin, Humboldt-Universität zu Berlin, Berlin, Germany; 4 Institute of Transfusion Medicine and Transplant Engineering, Hannover Medical School, Hannover, Germany; 5 Department of Biostatistics, Hannover Medical School, Hannover, Germany; 6 Department of Neurology, LMU University Hospital, Ludwig-Maximilians-University Munich, Munich, Germany; 7 Department of Neurology, Faculty of Medicine and University Hospital Cologne, University of Cologne, Cologne, Germany; 8 Department of Neurology, University Hospital Bonn, Bonn, Germany; 9 Allergy and Clinical Immunology Unit, Tel-Aviv Sourasky Medical Center, Tel-Aviv, Israel; 10 Department of Pediatric Hematology and Oncology, Hannover Medical School, Hannover, Germany; 11 German Center for Infection Research, Hannover, Germany; 12 German Center for Neurodegenerative Diseases, Munich, Germany; 13 Munich Cluster for Systems Neurology (SyNergy), Munich, Germany; 14 Centre for Individualised Infection Medicine, Hannover, Germany

## Abstract

**Importance:**

Progressive multifocal leukoencephalopathy (PML) is a life-threatening viral infection with no approved antiviral treatment.

**Objective:**

To determine whether restoring the compromised immune system of patients with PML with directly isolated allogeneic virus–specific (DIAVIS) T cells is a promising therapeutic strategy, especially if other curative options are absent.

**Design, Setting, and Participants:**

A retrospective case series of patients with PML who were treated with DIAVIS T cells was conducted between March 2020 and February 2022. T cells were isolated from healthy donors within 24 hours and targeted against the BK polyomavirus. Patients with PML were treated monocentrically. Eligibility for treatment with DIAVIS T cells was assessed for patients with confirmed PML, and exclusion criteria included stable PML disease and previous treatment with natalizumab.

**Exposure:**

Fresh DIAVIS T cells were administered with a maximum dose of 2 × 10^4^ CD3+ cells/kg body weight. Remaining T cells were cryopreserved in divided doses and administered in additional treatments approximately 2 and 6 weeks later.

**Main Outcomes and Measures:**

Primary outcome measures were clinical response and survival of patients, compared with the outcomes of a historical reference group of PML cases receiving best supportive treatment (BST) and with recently published real-world data of patients with PML who were treated with immune checkpoint inhibition.

**Results:**

The study cohort consisted of 28 patients (median [IQR] age, 60 [51-72] years; 20 male [71.4%]). Twenty-two patients (79%) treated with DIAVIS T cells showed response, resulting in significant clinical stabilization or improvement and a reduction in viral load. Six individuals (21%) were classified as nonresponders, deteriorated rapidly, and died, as did 2 other patients during a 12-month follow-up. Older age was the only predictor of a poor treatment response. Survival analysis revealed better 12-month survival rates (hazard ratio, 0.42; 95% CI, 0.24-0.73; *P* =.02) from diagnosis for patients treated with DIAVIS T cells (18 of 26 [69%]; 12-mo survival rate, 69%) compared with historical controls with BST (57 of 113 [50%]; 12-mo survival rate, including censored data, 45%).

**Conclusion and Relevance:**

This case series of DIAVIS T-cell therapy in PML provides first class IV evidence suggesting efficacy to reduce mortality and improve functional outcome. Further prospective studies are required to confirm these results.

## Key Points

**Question:**

Are directly isolated allogeneic virus–specific (DIAVIS) T cells associated with a therapeutic outcome in patients with progressive multifocal leukoencephalopathy (PML)?

**Findings:**

In this case series study including 28 patients with PML who were treated with DIAVIS T cells, 22 showed clinical response. Furthermore, survival analysis revealed significantly better 12-month survival rates from diagnosis for patients treated with DIAVIS T cells compared with best supportive treatment controls.

**Meaning:**

Results of this case series using DIAVIS T-cell therapy in PML provide first class IV evidence suggesting efficacy to reduce mortality and improve functional outcome in patients.

## Teaser

This case series investigates if directly isolated allogenic virus–specific T cells are associated with a therapeutic outcome in patients with progressive multifocal leukoencephalopathy (PML).

## Introduction

Progressive multifocal leukoencephalopathy (PML) is an opportunistic viral infection affecting gray and white matter cells of the brain caused by JC polyomavirus (JCV). Primary JCV infection, without causing PML, typically occurs in childhood, and antibodies against the virus remain detectable in up to 50% to 65% of adults.^1,2,3,4,5,6,7,8,9,10^ PML arises from genomic changes and reactivation of the virus, often triggered by compromised cellular immunity secondary to immune disorders or immunosuppressive medication.^11^ The severity of immunodeficiency influences the course of PML, particularly in lymphoproliferative disorders, leading to high mortality.^12,13^ Effective PML management requires restoration of immune competence. However, this cannot always be easily achieved, especially in conditions in which the endogenous immune competence is impaired by an underlying intrinsic process, eg, in lymphoproliferative disorders.^14^

Currently, there is no approved antiviral treatment for PML.^14^ Allogeneic JCV-specific T cells were first used to successfully treat a patient with PML in 2011.^15^ This therapeutic concept was further applied using BK polyomavirus (BKV)–specific T cells in 3 patients with PML in 2018 leading to improved neurological symptoms.^16^ Due to the high sequence homology of the 2 viruses,^17^ BKV-specific T cells may also be effective to treat PML, with widespread Good Manufacturing Practice (GMP) production already existing. Recently, feasibility of BKV-specific T-cell treatment for PML was shown in a phase 1 clinical study, albeit only 12 of 26 patients with PML underwent treatment, and several others died before receiving therapeutic approaches on a trial basis.^18^ To address the challenge of delay, we applied a novel approach with direct isolation of virus-specific T cells (VSTs) from partially human leukocyte antigen (HLA)–matched, related donors or unrelated donors from the unique T-cell donor registry (alloCELL) using the CliniMACS Cytokine Capture System (CCS) IFN-γ (Miltenyi Biotec).^19^ This manufacturing process facilitates a rapid availability of the VSTs within only 16 to 24 hours after leukapheresis, significantly accelerating the initiation of the therapeutic intervention by about 14 days. Here, we report the clinical outcomes of patients with PML who were treated with directly isolated allogeneic virus–specific (DIAVIS) T cells compared with 2 historical control groups receiving best supportive treatment (BST) or patients treated with immune checkpoint inhibition (ICI).

## Methods

### Study Outline

This case series involved a retrospective analysis of patients with confirmed PML who were treated with DIAVIS T cells at the Neurology Department of Hannover Medical School, Hannover, Germany. The treatment was carried out as compassionate use strictly following the corresponding ethical and legal framework of the Declaration of Helsinki, the German Arzneimittelgesetz, and the European Commission Directives. The study was approved by the local institutional review board. Every patient or their lawful representative provided written informed consent before inclusion. The Strengthening the Reporting of Observational Studies in Epidemiology (STROBE) reporting guideline has been followed, and the authors assume responsibility for the accuracy and completeness of the data and analyses.

### Patients

All included patients were adults over 18 years of age. All study participants self-identified as White race, and this information was collected at the beginning of treatment as part of their medical history. Currently, it is unknown whether race or ethnicity influences the course of PML. Patients were treated between March 2020 and February 2022. All patients had definite PML according to the American Academy of Neurology diagnostic criteria.^20^ The disease was progressive in all patients in terms of neurological symptoms and evolution of PML lesions on brain magnetic resonance imaging (MRI). Detailed information is provided in the eMethods in Supplement 1.

### Donor Selection and Clinical Grade Manufacturing of DIAVIS T Cells

Appropriate third-party donors were selected from at least 5/10 HLA partially matched first-degree relatives (family donors [FDs]) or unrelated individuals (unrelated donors [UDs]) from the alloCELL registry (Hannover, Germany) (eMethods and eFigures 1 and 2 in Supplement 1). HLA matching furthermore included at least 1 match each for HLA class I (A, B, C) and HLA class II (DR, DQ) to the patient in order to achieve an effect via HLA class I–restricted CD8^+^ cytotoxic T cells as well as via HLA class II–restricted CD4^+^ T helper cells. The potential donors were tested in advance for their frequencies of BKV-specific T cells using interferon (IFN)–γ cytokine secretion assay (CSA) and overlapping peptide pools against BKV VP1 and BKV LT antigens, as JCV-specific antigens were not available in terms of GMP at the time. CD3+/IFN-γ^+^ preenrichment ≥0.01% or enrichment of a clear, defined population of ≥10% CD3+/IFN-γ^+^ was accepted for clinical application. In case of a comparable HLA match, donors with higher BKV-specific T-cell frequencies were selected, whereas in case of comparable BKV-specific T-cell frequencies, donors with higher HLA match were preferred. Clinical-grade BKV-specific T cells were isolated from leukapheresis products of suitable donors using the CliniMACS Prodigy device (Miltenyi Biotec), the CliniMACS CCS system IFN-γ (Miltenyi Biotec), and GMP PepTivator BKV VP1 and BKV LT (Miltenyi Biotec). A single donor donated for 1 patient each. The detailed methodology is described in the eMethods in Supplement 1.

### Treatment With DIAVIS T Cells

Approximately 24 hours after leukapheresis, fresh DIAVIS T cells were administered with a maximum dose of 2 × 10^4^ CD3^+^ cells/kg body weight (eMethods and eFigure 2 in Supplement 1). The remaining T cells were cryopreserved in divided doses and administered in 1 or 2 additional treatments approximately 2 and 6 weeks after the first application depending on the number of cells obtained. Three patients received an additional fourth infusion due to clinical progression after the last T-cell dose, at intervals of 2, 3, and 4 months after the third T-cell application, respectively. Two of them responded; 1 patient did not respond.

### Clinical Follow-Up

The treatment was carried out within the framework of clinical routine procedures at the treating institution. The response to treatment was assessed at 6 weeks (median [IQR], 43 [40-48] days; minimum to maximum, 13-65 days), 3 months (median [IQR], 92 [84-100] days; minimum to maximum, 71-115 days), and 6 months (median [IQR], 185 [175-195] days; minimum to maximum, 98-281 days) after first cell transfer by clinical examinations including modified Rankin Scale (mRS) for Neurologic Disability, MRI (eFigure 8 in Supplement 1), and laboratory analyses of blood and cerebrospinal fluid (CSF). A detailed description is available in the eMethods in Supplement 1.

### Therapy Response and Survival Outcome Assessment

We compared therapy responders and nonresponders using mRS as the primary measure, evaluated by the same trained neurologists. Responders were defined as patients with stable or improved mRS score within the 6-month observational period and nonresponders as patients with a worsening mRS score. Additionally, we evaluated the survival rate 1 year after treatment for patients who received DIAVIS T cells and compared these results with data of historical patients.

### Historical Controls

Historical controls were categorized in 2 groups: patients who received BST (BST controls) and patients treated with experimental ICI (ICI controls). Both historical control groups closely matched with the DIAVIS T-cell–treated cohort regarding the causes of PML, as well as age and sex distribution. A detailed description is given in the eMethods in Supplement 1.

### Statistical Analysis

Statistical analyses were performed using GraphPad Prism, version 10 (GraphPad Software). The unpaired *t* test was used to compare continuous data for responders and nonresponders, and the Mann-Whitney test was applied for noncontinuous data. Two-way analysis of variance with Tukey multiple comparison test was used to compare the responders’ data at different time points (pretreatment vs week 6, month 3, and month 6). For nonresponders, the data from pretreatment were compared with those from week 6. The statistical comparison of the survival curves was carried out using the log-rank test. Results with a 2-sided *P* < .05 were considered statistically significant.

## Results

### Patient Treatment Selection Procedure

**Figure 1.**
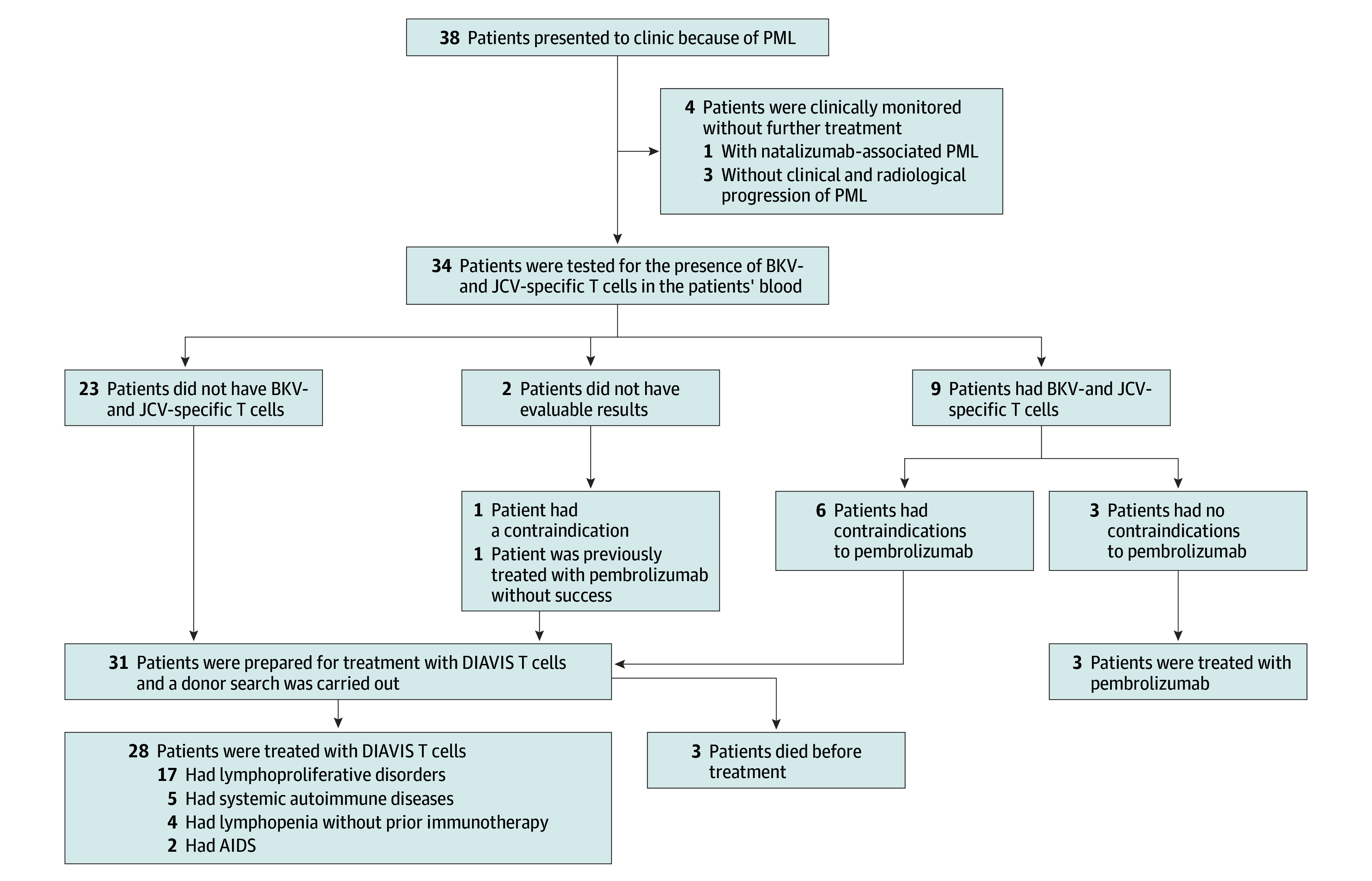
Description of the Procedures Followed for Patients Diagnosed With Progressive Multifocal Leukoencephalopathy (PML) BKV indicates BK polyomavirus; DIAVIS, directly isolated allogeneic virus specific; JCV, JC polyomavirus.

Thirty-eight patients were evaluated for potential PML treatment. After a stringent selection process (Figure 1 and eResults in Supplement 1), 28 patients (median [IQR] age, 60 [51-72] years; 8 female [28.6%]; 20 male [71.4%]) with progressive disease received DIAVIS T-cell treatment.

### Patient Characteristics

Table 1 and Table 2 (eResults and eTable 1 in Supplement 1) show detailed clinical characteristics of the 28 patients treated. The majority of patients with PML within our cohort were clinically severely impaired. At the time of first treatment, the median (IQR) mRS score was 4 (2-5). The most common leading clinical deficits were motor impairments with significant loss of mobility, aphasia, and/or dysarthria as well as qualitative and quantitative vigilance disturbances. The Montreal Cognitive Assessment (MoCA) test showed a median (IQR) score of 13 (0-23), indicating cognitive impairment in most patients.

**Table 1.** Clinical Characteristics of the Patients Treated With Directly Isolated Allogeneic Virus–Specific (DIAVIS) T Cells

Patient No.	Age at DIAVIS T-cell transfer	Sex	Underlying cause of PML	Category of underlying cause	Initial clinical deficits	mRS at DIAVIS T-cell transfer	Leading clinical deficits	No. of T-cell infusions	12-mo Survival	Time of death after first T-cell therapy, d
1	Mid-50s	F	Hodgkin disease, lung transplantation	Lympho proliferative	Disorientation, visual disturbances, headache, nausea	5	Quadriplegia, dysarthria, reduction of vigilance being bedridden	4	Alive	NA
2	Early 70s	F	Dermatomyositis, breast cancer	Autoimmune disease	Gait disturbance	5	Cerebellar syndrome being dependent on wheelchair, dysarthria, dysphagia with PEG-placement	4	Alive	NA
3	Mid 70s	M	Chronic lymphocytic leukemia	Lympho proliferative	Cognitive impairment	3	Left-sided hemiparesis being bedridden, dysphagia, cognitive impairment	1	Death due to PML	26
4	Mid 40s	M	Crohn disease	Autoimmune disease	Dysarthria, left-sided brachiofacial hemiparesis, headache, psychomotor deceleration	5	Reduction of vigilance being bedridden, dysarthria, left-sided brachiofacial hemiparesis, neglect left	3	Alive	NA
5	Mid 40s	M	Sarcoidosis	Autoimmune disease	Hypesthesia left hand, left-sided facial impairment	4	Left-sided spastic brachiofacial hemiparesis being bedridden, neglect left, cognitive impairment	3	Alive	NA
6	Early 80s	M	Chronic lymphocytic leukemia	Lympho proliferative	Cognitive impairment	3	Left-sided spastic brachiofacial hemiparesis being bedridden, neglect left, cognitive impairment	2	Death due to PML	43
7	Early 40s	M	Lymphopenia without preceding immunotherapy	Lymphopenia	Cognitive impairment, change of character, dysarthria	5	Reduction of vigilance in need of intensive care, quadriplegia	3	Alive	NA
8	Mid 50s	M	Lymphopenia without preceding immunotherapy	Lymphopenia	Dysarthria, left-sided facial impairment	2	Dysarthria, left-sided facial impairment, right-sided spastic arm paresis, spastic paraparesis	3	Alive	NA
9	Late 60s	M	Follicular lymphoma	Lympho proliferative	Aphasia	3	Dysarthria, aphasia, right-sided brachiofacial hemiparesis, right-sided hemihypesthesia, reduction of vigilance being bedridden	4	Death due to PML	139
10	Mid 50s	M	Pre-B acute lymphoblastic leukemia	Lympho proliferative	Right-sided arm paresis	5	Organic psychosyndrome being bedridden, right-dominant tetraparesis, dysarthria	3	Alive	NA
11	Mid 60s	M	Chronic lymphocytic leukemia	Lympho proliferative	Hypesthesia left hand	5	Organic psychosyndrome, severe reduction of vigilance being bedridden, spastic quadriplegia	2	Death due to PML	46
12	Early 50s	M	HIV infection	AIDS	Epileptic seizure	1	Epileptic seizures, tetraataxia, bradydysdiadochokinesa right arm	3	Alive	NA
13	Early 70s	M	Chronic lymphocytic leukemia	Lympho proliferative	Coordination disorder, dysarthria	3	Psychomotor deceleration, left-sided hemiparesis, neglect left	2	Alive	NA
14	Early 60s	M	Chronic lymphocytic leukemia	Lympho proliferative	Visual disturbances	5	Cortical blindness, reduction of vigilance being bedridden, apraxia, dysarthria, cognitive impairment, dysphagia with PEG and tracheotomy placement	3	Alive	NA
15	Early 60s	M	Diffuse large B-cell lymphoma	Lympho proliferative	Aphasia, confusion	3	Sensomotoric aphasia, apraxia, left-sided hemiparesis	3	Death due to PML	203
16	Late 70s	M	Diffuse large B-cell lymphoma	Lympho proliferative	Fine motor dysfunction of right hand, latent right-sided leg paresis	4	Right-sided hemiparesis, right-sided hemihypesthesia, gait ataxia, motor aphasia	2	Alive	NA
17	Late 50s	F	Early T acute lymphoblastic leukemia	Lympho proliferative	Left-sided leg paresis	4	Left-sided brachiofacial hemiparesis being bedridden, psychomotor deceleration	3	Alive	NA
18	Mid 50s	M	HIV infection	AIDS	Gait disturbance	4	Right-sided hemiparesis, visual disturbance, ataxia, motor aphasia	3	Alive	NA
19	Early 80s	M	Chronic lymphocytic leukemia	Lympho proliferative	Aphasia	5	Right-sided hemiplegia being bedridden, neglect right, aphasia	3	Death due to PML	72
20	Late 50s	M	Multiple myeloma	Lympho proliferative	Left-sided arm paresis, hypesthesia left arm	4	Left-sided hemiparesis being dependent on wheelchair, left-sided hemihypesthesia, neglect left	2	Alive	NA
21	Early 80s	M	Kidney failure, dialysis-dependent	Lymphopenia	Cognitive impairment, left-sided hemiparesis, reduction of general condition	5	Cognitive impairment, left-sided hemiparesis being bedridden	2	Death due to COVID-19	155
22	Early 30s	M	Acute lymphoblastic leukemia	Lympho proliferative	Right-sided leg paresis	4	Left-sided hemiparesis being dependent on wheelchair	3	Alive	NA
23	Early 80s	F	Rheumatoid arthritis	Autoimmune disease	Disorientation, change of character	5	Reduction of vigilance, left-sided hemiparesis being bedridden, neglect left	3	Death due to age	222
24	Early 40s	F	Hodgkin lymphoma, diffuse large B-cell lymphoma	Lympho proliferative	Headache	3	Cephalgia, bilateral visual reduction, left-sided homonymous hemianopsia, cognitive impairment	3	Alive	NA
25	Late 60s	F	Follicular lymphoma	Lympho proliferative	Cognitive impairment	4	Aphasia, cognitive impairment, right-sided hemiparesis	2	Alive	NA
26	Early 70s	F	Cutaneous polyarteritis nodosa	Autoimmune disease	Aphasia, left-sided arm paresis, left-sided hemihypesthesia, neglect left	3	Aphasia, apraxia, organic psychosyndrome, left-sided arm paresis, left-sided hemihypesthesia, neglect left	3	Alive	NA
27	Mid 60s	F	Follicular lymphoma	Lympho proliferative	Headache, visual disturbance, gait disturbance, dizziness	2	Gait ataxia, hypermetric saccades, depressive episode	3	Alive	NA
28	Late 30s	M	Severe combined immunodeficiency	Lymphopenia	Aphasia	3	Motor aphasia, apraxia	3	Alive	NA

Abbreviations: mRS, modified Rankin Scale; NA, not applicable; PEG, percutaneous endoscopic gastrostomy; PML, progressive multifocal leukoencephalopathy.

**Table 2.** Characteristics of the Patients Pretreatment

Characteristics	All patients with PML (n = 28)	Responder (n = 22)	Nonresponder (n = 6)	Comparison responder vs nonresponder	
				Difference (95% CI)	*P* value
Age, median (IQR), y	60 (51 to 72)	57 (46 to 71)	71 (65 to 82)	14.14 (1.58 to 26.69)	.03
Female/male, No. (%)	8 (28.6)/20 (71.4)	8 (36.4)/14 (63.6)	0/6 (100)	0.0 (−1.0 to 0.0)	.14
Disability at onset of therapy					
mRS, median (IQR)	4 (3 to 5)	4 (3 to 5)	3 (3 to 5)	−0.1515 (−1.264 to 0.9607)	.76
MoCA, median (IQR)^a^	13 (0 to 23)	17 (3 to 26)	1 (0 to 15)	−9.438 (−21.98 to 3.109)	.13
Days of disease progression to treatment, median (IQR)	90 (49 to 128)	80 (49 to 111)	114 (43 to 157)	13.15 (−36.74 to 63.04)	.59
Underlying disease					
Lymphoproliferative disorders, No.	17 (61)	11 (50)	6 (100)	0.50 (0.0 to 1.0)	.05
Autoimmune diseases, No.	5 (18)	5 (23)	0 (0)	0.0 (0.0 to 0.0)	.32
Lymphopenia, No.	4 (14)	4 (18)	0 (0)	0.0 (0.0 to 0.0)	.55
AIDS, No.	2 (7)	2 (9)	0 (0)	0.0 (0.0 to 0.0)	>.99
Previous CD20 depletion, No. (%)	11 (39)	7 (32)	4 (67)	1.0 (0.0 to 1.0)	.17
Previous stem cell therapy, No. (%)	8 (29)	6 (27)	2 (33)	0.0 (0.0 to 1.0)	>.99
Autologous, No. (%)	4 (50)	3 (50)	1 (50)	0.0 (0.0 to 0.0)	>.99
Allogeneic, No. (%)	4 (50)	3 (50)	1 (50)	0.0 (0.0 to 0.0)	>.99
MRI of the brain–lesion description					
Classic PML, No. (%)	10/27 (37)	9/21 (43)	1/6 (17)	0.0 (−1.0 to 0.0)	.36
Inflammatory PML, No. (%)	17/27 (63)	12/21 (57)	5/6 (83)	0.0 (0.0 to 1.0)	.36
MRI of the brain–lesion spread					
Unifocal, No. (%)	0/28 (0)	0/22 (0)	0/6 (0)	0.0 (0.0 to 0.0)	>.99
Multifocal, No. (%)	3/28 (11)	2/22 (9)	1/6 (17)	0.0 (0.0 to 0.0)	>.99
Widespread, No. (%)	25/28 (89)	20/22 (91)	5/6 (83)	0.0 (0.0 to 0.0)	>.99
Blood parameters^a^					
Leukocytes/μL, median (IQR)	6800 (4350 to 11 750)	6500 (4075 to 8025)	43 700 (4950 to 196 425)	76 524 (35 342 to 117 707)	<.001
Lymphocytes/μL, median (IQR)	1240 (608 to 3225)	1038 (580.5 to 2071)	17 408 (1549 to 110 845)	43 980 (10 956 to 77 004)	.01
CD4^+^ cells/μL, median (IQR)	249 (148 to 663)	241 (132.5 to 526.5)	1109 (244 to 2895)	1059 (292.4 to 1826)	.01
CD8^+^ cells/μL, median (IQR)	511 (232 to 1428)	280 (153 to 1045)	1671 (857 to 2380)	978.3 (82.51 to 1874)	.03
CD20^+^ cells/μL, median (IQR)	85 (1.75 to 441)	70 (1.75 to 302)	33 415 (3351 to 131 240)	54 147 (19 221 to 89 073)	.01
Cerebrospinal fluid parameters					
Cell count/μL, median (IQR)	3 (1 to 7)	3 (1 to 8)	1 (1 to 6)	−1.471 (−5.65 to 2.71)	.48
Total protein mg/dL, median (IQR)	469 (407 to 579)	469 (415 to 573)	533 (345 to 692)	37.74 (−74.35 to 149.8)	.49
Albumin ratio, median (IQR)	7.68 (5.09 to 9.04)	7.68 (5.28 to 8.63)	7.68 (4.67 to 10.81)	0.60 (−1.81 to 3.02)	.61
Oligoclonal bands positive (%)	74	81	50	0.0 (−1.0 to 0.0)	.29
Viral load copies/mL, median (IQR)	1900 (500 to 8683)	1900 (500 to 8228)	4500 (575 to 81 750)	47 769 (−3051 to 98 588)	.06
tTau pg/mL, median (IQR)	363 (231 to 692.5)	308 (196.5 to 588)	573 (289 to 897.5)	168.8 (−179.0 to 516.6)	.32
pNfH pg/mL, median (IQR)	1845 (1452 to 3645)	1857 (1389 to 3437)	1823 (1457 to 7718)	750.4 (−2776 to 4277)	.66

Abbreviations: MoCA, Montreal Cognitive Assessment; MRI, magnetic resonance imaging; mRS, modified Rankin Scale; PML, progressive multifocal leukoencephalopathy; pNfH, phosphorylated neurofilament heavy chain; tTau, total tau protein.

SI conversion factor: To convert leukocytes and lymphocytes to ×10^9^/L, multiply by 0.001.

^a^ Data not available for all patients.

### Donor Selection and Manufacturing of DIAVIS T Cells

In 9 patients, the T-cell donors were first-degree family members with an HLA match between 5/10 to 10/10 (eFigure 2 in Supplement 1). No suitable partially HLA-matched family members were available for 19 patients. For these, unrelated third-party donors from the alloCELL registry were chosen whose HLA match was 5/10 to 8/10. The median (IQR) time between donor search and final donor clearance was 7 (2-11) days. The subsequent median (IQR) time to production of DIAVIS T cells required 9 (5-20) days (eFigure 2 in Supplement 1). There were no significant differences between FD and UD in the days required of selection (difference, 0.003; 95% CI, −4.06 to 5.07; *P* = .58) or subsequent manufacturing of VSTs (difference, −1.01; 95% CI, −12.17 to 3.04; *P* = .43). The proportion of IFN-γ^+^-VSTs in the T-cell product was similar between FD and UD in terms of CD3^+^ VSTs (difference, −0.17; 95% CI, −6.59 to 6.25; *P* = .17), CD8^+^ VSTs (difference, −3.21; 95% CI, −11.41 to 4.98; *P* = .21), and CD4^+^ VSTs (difference, 0.48; 95% CI, −6.07 to 7.02; *P* = .21) (eFigure 2 in Supplement 1). The VSTs secrete IFN-γ on exposure to the viral peptide pools. Therefore, the frequency of IFN-γ^+^ T cells among the CD3^+^ (or CD8^+^/CD4^+^) T cells (eFigures 1 and 2 in Supplement 1) correspond to the number of VSTs. The median number of transferred cells was 5.75 × 10^4^ CD3^+^ T cells/kg body weight (range, 1.0-6.0 × 10^4^) and 1.36 × 10^4^ IFN-γ+CD3^+^ T cells/kg body weight (range, 0.18-3.21 × 10^4^).

### Responder Analysis

During the 6-month follow-up, 22 patients (79%) were classified as responders (ie, mRS scores stabilized in 12 patients and improved in 10 patients). The remaining 6 patients (21%) deteriorated in their mRS score (eFigure 7 in Supplement 1) and were designated as nonresponders, all of whom later died of PML, as did 2 other patients during a 12-month follow-up (eResults and eTable 2 in Supplement 1).

**Figure 2.**
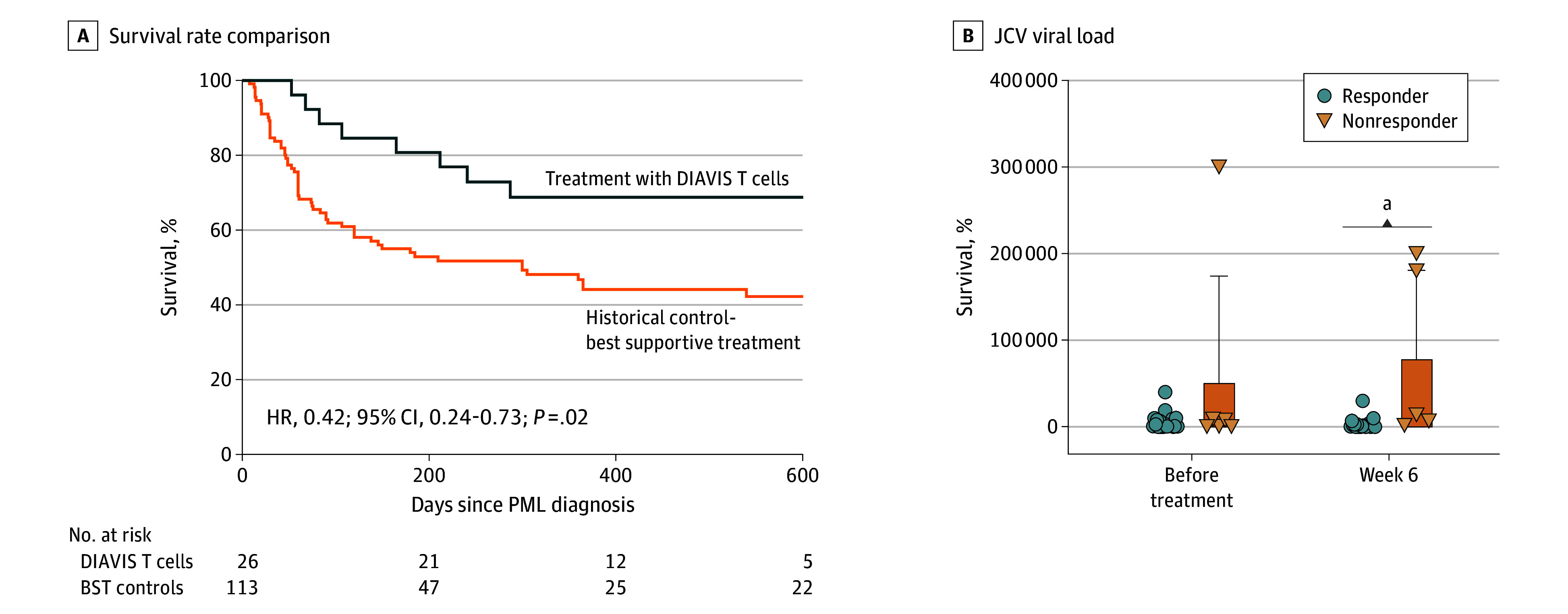
Comparison of Survival Rates Against Best Supportive Treatment (BST) Controls and Comparative Analysis of Responders and Nonresponders Treated With Directly Isolated Allogeneic Virus–Specific (DIAVIS) T Cells A, Comparison of the survival curves of patients treated with DIAVIS T cells with those of historical controls who received BST. B, Cerebrospinal fluid (CSF) values of JC polyomavirus (JCV) viral load in treatment responders and nonresponders comparing the changes observed in viral load from pretreatment to week 6 in both groups showing mean with SD. Week 6 = median day 43 (IQR, 40-48 days; minimum to maximum, 13-65 days). HR indicates hazard ratio; PML, progressive multifocal leukoencephalopathy. ^a^*P* =.004

Six weeks after initiation of therapy, no significant differences between responders and nonresponders were observed in terms of mRS score (eFigure 3 in Supplement 1) and CSF levels of phosphorylated neurofilament heavy chain (NfH), a marker of neuronal damage^21,22^ (eFigure 3 in Supplement 1). However, there were statistically significant differences in the change of the pretreatment and posttreatment mRS scores (difference, 0.5; 95% CI, 0-2.0; *P* = .01) (eFigure 3 in Supplement 1) and phosphorylated NfH levels (difference, 1938 pg/mL; 95% CI, 252-11 163 pg/mL; *P* = .04) (eFigure 3 in Supplement 1) between the groups. At week 6, responders had a notably lower viral load compared with nonresponders (Figure 2) (difference, −77 421 copies/mL; 95% CI, −132 676 to −22 166 copies/mL; *P* = .01). Although trends showed decreasing viral load from pretreatment to week 6 for responders and increasing load for nonresponders within the same period of time, this was not statistically significant (eFigure 3 in Supplement 1).

During the 6-week follow-up, 16 of 22 therapy-responsive patients (72.7%) had detectable human polyomavirus (HPV)–specific T cells in their blood (eFigures 3 and 4 in Supplement 1). One of the nonresponders (6 of 28), who had received 1 dose of DIAVIS T cells and later died, had detectable HPV-specific T cells. Donor cell source did not affect treatment outcomes, as 79% of patients (15 of 19) and 78% of patients (7 of 9) who received DIAVIS T cells from UDs and FDs, respectively, responded (eFigure 3 in Supplement 1). In addition, responders and nonresponders did not differ in the number of T-cell doses. Both groups received a median (IQR) of 3 (2-3) doses. Furthermore, the total administered cell dose (CD3^+^ T cells) and the total number of administered specific CD3^+^IFN-γ^+^ T cells did not differ between responders and nonresponders (eFigure 5 in Supplement 1).

### Survival Analysis

All six nonresponders (21%) died of PML-related neurological deterioration within a median (IQR) of 59 (44-122) days after treatment initiation. Of the 22 responders, 1 patient improved from PML but died of severe COVID-19 infection 212 days after diagnosis, and another improved but died 241 days postdiagnosis at the age of 81 years. The median (IQR) duration from PML diagnosis to death was 136 (79-219) days.

The BST-control group included patients with lymphoproliferative disorders, systemic autoimmune diseases, and lymphopenia, similar to our treated cohort. For survival analysis, AIDS-related PML cases from the historical control group and 2 patients positive for HIV who responded to DIAVIS T cells were excluded, as, according to the literature, these patients tend to show better survival than other patients with PML, and in many cases, PML can already be positively influenced by highly active antiretroviral therapy.^23^ Due to the similar distribution of the underlying diseases and the identical median age of 60 years in both cohorts, it can be assumed that the BST-control group and the cohort of patients treated with DIAVIS T cells were meaningfully similar in terms of PML prognosis. In the BST-control group, which comprised 113 patients (eResults and eTables 2 and 3 in Supplement 1), 57 patients died of PML within 1 year, with a median (IQR) survival of 60 (30-114) days. Survival analysis revealed a significant benefit (hazard ratio [HR], 0.42; 95% CI, 0.24-0.73; *P* = .02) for patients with PML who are treated with DIAVIS T cells (18 of 26 [69%]; 12-mo survival rate, 69%) compared with this group (57 of 113 [50%]; 12-mo survival rate, including censored data, 45%) (Figure 2).

The second historical reference group consisted of 67 patients who were experimentally treated with ICI.^24^ Their underlying causes of PML and the median age of 65 years closely mirrored those of our treated cohort. The similar distribution of underlying diseases and ages in this cohort implies a comparable prognosis with the T-cell–treated group. Within 1 year, 56% of this group (38 patients) died. A comparison with patients with PML who were treated with DIAVIS T cells showed no significant difference between the 2 groups (log-rank test: HR, 0.55; 95% CI, 0.28-1.06; *P* = .12) (eFigure 6 in Supplement 1).

### Prognostic Factors

The comparison of pretreatment prognostic characteristics in responders and nonresponders is shown in Table 2. Nonresponders were significantly older than responders (difference, 14.1 years; 95% CI, 1.58-26.69 years; *P* = .03), no significant differences were found in other clinical, CSF, and MRI characteristics. Nonresponders had higher white blood cell counts, primarily due to the inclusion of patients with chronic lymphocytic leukemia with lymphocytosis. Donor-related factors, such as donor source and the number of matched HLA alleles as well as the characteristics of infused DIAVIS T cells, had no significant influence on treatment response (eFigure 2 in Supplement 1).

### Outcome Parameters in Responders

The clinical and laboratory parameters of the responders improved continuously during the follow-up (Table 3). Within 3 months, disability decreased significantly (mRS score difference pretreatment to third month, 0.47; 95% CI, 0.08-0.86; *P* = .01), with further improvement at 6 months. Cognitive function also improved, with a significant increase in MoCA scores after 6 months as compared with pretreatment (difference, −3.57; 95% CI, −6.18 to −0.96; *P* = .001).

**Table 3.** Outcome Parameters of Responders During Follow-Up^a^^,^^b^

Parameters	Before treatment	Week 6			Month 3			Month 6		
		Absolute	Difference (95% CI)	*P* value	Absolute	Difference (95% CI)	*P* value	Absolute	Difference (95% CI)	*P* value
Clinical status										
Modified Rankin Scale, median (IQR)	4 (3 to 5)	4 (3 to 5)	0.27 (−0.11 to 0.66)	.28	3 (2 to 4)	0.47 (0.08 to 0.86)	.01	3 (2 to 4)	0.78 (0.39 to 1.18)	<.001
MoCA, median (IQR)^a^	17 (3 to 26)	17 (2 to 27.5)	−1.51 (−4.18 to 1.17)	.44	21 (8 to 26)	−2.15 (−4.7 to 0.4)	.12	22 (11 to 25)	−3.57 (−6.18 to −0.96)	.01
MRI of the brain										
T2 lesion load reduction, No./total No. (%)	NA	5/21 (24)	−0.24 (−0.49 to 0.01)	.07	10/18 (56)	−0.29 (−0.56 to −0.02)	.03	9/19 (47)	0.07 (−0.2 to 0.34)	.91
T2 lesion load increase, No./total No. (%)	NA	8/21 (38)	−0.38 (−0.61 to −0.15)	<.001	3/18 (17)	0.25 (−0.0003 to 0.49)	.05	3/19 (16)	−0.002 (−0.25 to 0.25)	>.99
Gd + enhancement present, No./total No. (%)	9/21 (43)	10/21 (52)	−0.05 (−0.19 to 0.09)	.8	8/18 (44)	0.12 (−0.04 to 0.26)	.23	10/19 (47)	−0.11 (−0.26 to 0.04)	.22
Cerebrospinal fluid										
Cell count/μL, median (IQR)	3 (1 to 8)	2 (1 to 6)	0.79 − 1.32 to 2.91	.75	2 (1 to 4)	1.79 (−0.36 to 3.95)	.14	2 (1 to 3)	3.16 (0.97 to 5.36)	.01
Total protein mg/dL, median (IQR)	469 (415 to 573)	500 (427 to 563)	7.48 (−40.53 to 55.48)	.98	445 (393 to 555.5)	22.94 (−25.17 to 71.05)	.59	459 (351 to 555)	41.03 (−7.925 to 89.99)	.13
Albumin ratio, median (IQR)	7.68 (5.28 to 8.63)	7.52 (6.25 to 9.40)	−0.09 (−1.140 to 0.97)	>.99	6.65 (5.14 to 8.84)	−0.04 (−1.11 to 1.04)	.99	6.50 (4.85 to 8.74)	0.56 (−0.53 to 1.65)	.53
Oligoclonal bands positive	81	86	−0.05 (−0.33 to 0.23)	.96	86	−0,05 (−0.33 to 0.23)	.96	86	−0.02 (−0.31 to 0.27)	>.99
JCV load, copies/mL, median (IQR)	1900 (500 to 8228)	1000 (15 to 3000)	2488 (−1052 to 6028)	.26	167 (0 to 983)	4056 (449.7 to 7661)	.02	300 (0 to 1000)	5057 (1387 to 8726)	.01
JCV present, PCR positivity, No./total No. (%)	20/22 (91)	16/21 (76)	0.19 (−0.06 to 0.45)	.21	13/20 (65)	0.35 (0.09 to 0.60)	.01	10/19 (53)	0.49 (0.23 to 0.76)	<.001
tTau pg/mL, median (IQR)	308 (196.5 to 588)	264 (188 to 549)	−38.40 (−196.9 to 120.1)	.92	268 (181 to 344)	133.3 (−25.56 to 292.2)	.13	208 (182.5 to 245)	148.6 (−14.10 to 311.3)	.08
pNfH pg/mL, median (range)	1857 (1389 to 3437)	1614 (1141 to 2489)	1062 (−559.3 to 2684)	.31	1332 (810 to 2137)	1929 (100.9 to 3756)	.04	788 (509 to 1293)	1703 (−124.9 to 3530)	.08
Virus-specific T cells										
BKV/JCV-specific T cells detectable, No./total No. (%)	7/20 (35)	16/22 (73)	−0.43 (−0.68 to −0.18)	<.001	12/18 (66)	−0.24 (−0.51 to 0.02)	.09	11/18 (61)	−0.19 (−0.45 to 0.08)	.26
BKV-specific T cells detectable, No./total No. (%)	3/20 (15)	11/22 (50)	−0.38 (−0.63 to −0.13)	<.001	8/18 (44)	−0.25 (−0.51 to 0.02)	.08	4/18 (22)	−0.02 (−0.29 to 0.24)	>.99
JCV-specific T cells detectable, No./total No. (%)	6/20 (30)	16/22 (73)	−0.48 (−0.73 to −0.22)	<.001	12/18 (66)	−0.29(−0.57 to −0.02)	.03	11/18 (61)		.11
Blood leukocytes^a^										
Leukocytes/μL, median (IQR)	6500 (4075 to 8025)	5700 (4300 to 7300)	−433.0 (−3078 to 2212)	.97	6000 (5100 to 8250)	789.6 (−1741 to 3320)	.84	6850 (4775 to 7700)	325.6 (−2254 to 2905)	.99
Lymphocytes/μL, median (IQR)	1038 (580.5 to 2071)	912 (597.5 to 1812)	−683.7 (−3439 to 2071)	.91	1125 (512.5 to 2160)	259.4 (−2328 to 2847)	.99	1036 (677 to 2618)	1239 (−1430 to 3908)	.6
CD4+ cells/μL, median (IQR)	241 (132.5 to 526.5)	287 (62 to 428)	−16.12 (−146.5 to 114.2)	.99	289 (99.5 to 588)	−93.17 (−209.7 to 23.41)	.15	321 (156 to 374.5)	−32.55 (−158.2 to 93.06)	.89
CD8+ cells/μL, median (IQR)	280 (153 to 1045)	262 (119 to 500)	−34.45 (−207.9 to 139.0)	.95	416 (115 to 1347)	−36.42 (−191.5 to 118.7)	.92	392 (134.5 to 1218)	−46.30 (−213.5 to 120.9)	.87
CD20+ cells/μL, median (IQR)	70 (1.75 to 302)	120 (35 to 178)	−937.4 (−3933 to 2058)	.83	162 (27.5 to 272)	151.8 (−2527 to 2831)	.99	180 (41 to 466.5)	1035 (−1852 to 3921)	.76

Abbreviations: BKV, BK polyomavirus; JCV, JC polyomavirus; MoCA, Montreal Cognitive Assessment; PCR, polymerase chain reaction; pNfH, phosphorylated neurofilament heavy chain; tTau, total tau protein.

SI conversion factor: To convert leukocytes and lymphocytes to ×10^9^/L, multiply by 0.001.

^a^ Data not available for all patients. Difference (95% CI) as difference to pretreatment calculation (exception: magnetic resonance imaging of the brain as calculation of difference to previous time point).

^b^ Clinical and MRI parameters were compared to their pretreatment condition at different time points. Week 6 = median, 44 days (IQR, 41-49 days; minimum to maximum, 29-65 days), month 3 = median, 94 days (IQR, 84-101 days; minimum to maximum, 71-115 days), month 6 = median, 187 days (IQR, 179-195 days; minimum to maximum, 165-281 days).

CSF viral load decreased significantly within 3 months (difference pretreatment to third month, 4056 copies/mL; 95% CI, 449.7-7661 copies/mL; *P* = .02), with a further decrease after 6 months. Remarkably, no JCV DNA was detectable in the CSF of 9 patients after 6 months. The pretreatment levels of the CNS neuronal damage marker phosphorylated NfH decreased significantly (difference pretreatment to third month, 1929 pg/mL; 95% CI, 100.9-3756 pg/mL; *P* = .04) by the third month. Standard CSF parameters remained stable, except for a significant decrease in CSF cell count after 6 months (difference, 3.16 cells/μL; 95% CI, 0.97-5.36 cell/μL; *P* = .002). Blood leukocyte and lymphocyte counts, as well as subpopulation markers (CD4^+^, CD8^+^, CD20^+^), remained unchanged.

Analysis of VST via enzyme-linked immunospot (ELISpot [Lophius Biosciences]) assay (eMethods in Supplement 1) revealed a significant increase for BKV/JCV–specific T cells (difference, −0.43; 95% CI, −0.68 to −0.18; *P* < .001) and each subpopulation (JCV: difference, −0.48; 95% CI, −0.73 to −0.22; *P* < .001; BKV: difference, −0.38; 95% CI, −0.63 to −0.13; *P* < .001), respectively, at week 6 (Table 2).

MRI analysis revealed a significant increase in T2 lesion load pretreatment to week 6 (difference, −0.38; 95% CI. −0.61 to −0.15; *P* < .001) and a decrease in T2 lesion load from week 6 to month 3 (difference, −0.29; 95% CI, −0.56 to −0.02; *P* = .03) (Table 2). No significant changes were observed in gadolinium enhancement over time.

### Tolerability

All patients tolerated the therapy well with only minor adverse effects, including a transient skin rash in 3 patients, a short-term increase in liver enzymes with reddening of the palms in 1 patient, and probable transient infectious diarrhea in another. All symptoms resolved spontaneously within a few days and were not related to graft-vs-host disease (GVHD).

Three patients experienced a transient slight decline of their clinical condition, associated with new or increased contrast-enhancing lesions on MRI, suggesting an immune response in terms of an immune reconstitution inflammatory syndrome. One patient received high-dose intravenous steroids for 3 days, and the others were closely monitored. Notably, all 3 patients responded well to treatment and showed improvements in subsequent evaluations.

## Discussion

We report an innovative therapeutic approach for PML, a disease with high mortality and lack of effective antiviral therapies. Results of this case series of 28 patients with PML treated with DIAVIS T cells demonstrates the therapy’s significant potential, as 22 patients responded and 20 survived beyond 12 months.

Results suggest that our method, inspired by adoptive transfer of VSTs to treat posttransplant viral complications,^15,25^ appears particularly suitable for patients with PML who lack endogenous antiviral T-cell activity. In a 2021 study, 12 patients with PML received donor-derived, BKV-specific T cells, 7 of whom survived for 12 months.^18^ However, the 4- to 6-week time required to obtain a final clinical T-cell product led to treatment delays and the exclusion of 6 rapidly deteriorating patients. Our direct isolation approach addressed these limitations and significantly reduced the time of cell generation from 14 days in the aforementioned studies to 16 to 24 hours and the entire process of providing the clinical T-cell product to less than 3 weeks. Additionally, our approach may minimize the potential risk of GVHD by focusing on BKV memory T cells and limiting T-cell doses to a maximum of 20 000 CD3^+^ T-cells/kg body weight, which is in line with safe limits for donor lymphocyte infusion in HLA haploidentical settings. Consistently, significantly reduced lymphocyte proliferation was reported in the absence of naive T cells in mismatched settings with VST enrichment.^26,27^ Notably, in our cohort, only mild and transient skin reaction occurred in 3 patients. Thus, the treatment-related adverse events were generally in line with those in the previously published studies on the use of VSTs in PML.^16,18^ Furthermore, we found that the donor cell source or HLA allele-match distribution had no impact on treatment success, being similar in responders and nonresponders. This broadens the donor pool and speeds up matching for patients without suitable FDs, which was the case for approximately two-thirds of patients.

In our cohort, 22 patients rapidly responded to treatment, whereas 6 deteriorated quickly. A comparison of responders and nonresponders showed significant differences in the change in clinical scores and phosphorylated NfH levels as a marker for neuronal damage before and 6 weeks after therapy. Unfortunately, nonresponders could not be compared after 3 months due to rapid mortality. However, responders demonstrated significant clinical improvement, correlating with reduced CNS viral load and phosphorylated NfH levels.

Effective viral control in PML relies on the ability of the infused T cells to recognize and eliminate infected cells while also promoting the patient’s own VST response. This dual action requires a partial HLA match to optimize CD4^+^ and CD8^+^ T-cell synergy for efficient virus clearance.^28^ In our study, all but 1 of the nonresponsive patients lacked their own VSTs detectable in the blood 6 weeks after starting treatment with DIAVIS T cells in contrast to most responders. Despite using BKV-specific T cells to treat PML caused by JCV, significant cross-reactivity occurs due to the substantial protein similarity between the VP1 (78%) and LT (83%) proteins of these viruses.^17^ In our cohort, we observed that the administration of BKV-specific T cells also led to the detection of JCV-specific T cells in the blood of responders after 6 weeks of treatment. This outcome suggests that the therapy notably improves the patients’ natural ability to generate VSTs. However, it can also be hypothesized that therapy with allogeneic JCV-specific T cells could possibly lead to a further improvement in the outcome of patients with PML. In addition, it would be very valuable to track the transferred T cells and their persistence in the patients by chimerism analyses. This could also shed light on the significance of the HLA match in relation to the CD8^+^ and CD4^+^ T-cell responses. This should be investigated in future studies.

We compared our results with a historical control group of patients with PML with similar underlying causes who did not receive experimental T-cell therapy or ICI. Patients treated with DIAVIS T cells showed significantly better survival rate compared with the BST controls. The comparison with the second control group of ICI-treated patients showed a trend toward better survival rates in our cohort compared with published data on ICI treatment. The lower survival rate in patients treated with ICI may be due to the absence of endogenous VSTs, a pattern also seen in our cohort in which most lacked these T cells before therapy. This suggests a limited efficacy of ICI in the absence of VSTs. Of note, 2 of our patients treated with DIAVIS T cells who were previously treated with ICI continued to have PML progression and had no detectable VSTs.

### Limitations

This study has some limitations. In our cohort, patients were treated regardless of the severity of their disease or their clinical status. It is important to emphasize that our analysis was not part of a clinical study with prespecified inclusion/exclusion criteria and outcome measures. To establish more robust evidence, conducting a study using such treatment under standard conditions will be required and is currently being designed.

## Conclusions

This case series of DIAVIS T-cell therapy in patients with PML provides first class IV evidence suggesting efficacy to reduce mortality and improve functional outcome. Further prospective studies are required to confirm these results.

## Notes

Supplement 1.
eMethods.

eResults.
**eFigure 1.** Overview of Donor-Selection and the Manufacturing Process of DIAVIS T Cells **eFigure 2.** Comparative Analysis for Cell Manufacturing Processes and Product Quality Based on Donor Source **eFigure 3.** Comparative Analysis of Responders and Nonresponders **eFigure 4.** Individual Immunomonitoring in Patients Pretreatment and 6 Weeks Posttreatment With DIAVIS T Cells **eFigure 5.** Correlation Between Cellular Composition, Purity, and Cell Dose in Responders (R) vs Nonresponders (NR) **eFigure 6.** Comparison of Survival Rate Against Historical Control of Immune Checkpoint Inhibition **eFigure 7.** mRS Development of Nonresponders During the Observation Period **eFigure 8.** Monitoring and Classifying Lesion on MRI **eTable 1.** Additional Characteristics of the Patients Treated With DIAVIS T Cells **eTable 2.** Detailed Characteristics of Best Supportive Treatment Controls **eTable 3.** Detailed Characteristics of Best Supportive Treatment Controls Published Within the Literature
eReferences

Supplement 2.
Data Sharing Statement.

## References

[1] Major EO, Amemiya K, Tornatore CS, Houff SA, Berger JR. Pathogenesis and molecular biology of progressive multifocal leukoencephalopathy, the JC virus-induced demyelinating disease of the human brain. Clin Microbiol Rev. 1992;5(1):49-73. doi:10.1128/CMR.5.1.49 1310438 PMC358223

[2] Knowles WA, Pipkin P, Andrews N, . Population-based study of antibody to the human polyomaviruses BKV and JCV and the simian polyomavirus SV40. J Med Virol. 2003;71(1):115-123. doi:10.1002/jmv.10450 12858417

[3] Monaco MC, Atwood WJ, Gravell M, Tornatore CS, Major EO. JC virus infection of hematopoietic progenitor cells, primary B lymphocytes, and tonsillar stromal cells: implications for viral latency. J Virol. 1996;70(10):7004-7012. doi:10.1128/jvi.70.10.7004-7012.1996 8794345 PMC190751

[4] Warnke C, Ramanujam R, Plavina T, . Changes to anti-JCV antibody levels in a Swedish national MS cohort. J Neurol Neurosurg Psychiatry. 2013;84(11):1199-1205. doi:10.1136/jnnp-2012-304332 23463870 PMC3812878

[5] Trampe AK, Hemmelmann C, Stroet A, . Anti-JC virus antibodies in a large German natalizumab-treated multiple sclerosis cohort. Neurology. 2012;78(22):1736-1742. doi:10.1212/WNL.0b013e3182583022 22592369

[6] Bonek R, Guenter W, Jałowiński R, . JC Virus Seroprevalence and JCVAb index in Polish multiple sclerosis treatment-naive patients. J Clin Med. 2020;9(12):3867. doi:10.3390/jcm9123867 33261210 PMC7759948

[7] Olsson T, Achiron A, Alfredsson L, . Anti-JC virus antibody prevalence in a multinational multiple sclerosis cohort. Mult Scler. 2013;19(11):1533-1538. doi:10.1177/1352458513477925 23459571

[8] Tan CS, Dezube BJ, Bhargava P, . Detection of JC virus DNA and proteins in the bone marrow of HIV-positive and HIV-negative patients: implications for viral latency and neurotropic transformation. J Infect Dis. 2009;199(6):881-888. doi:10.1086/597117 19434914 PMC2893283

[9] Egli A, Infanti L, Dumoulin A, . Prevalence of polyomavirus BK and JC infection and replication in 400 healthy blood donors. J Infect Dis. 2009;199(6):837-846. doi:10.1086/597126 19434930

[10] Schweitzer F, Laurent S, Cortese I, . Progressive multifocal leukoencephalopathy: pathogenesis, diagnostic tools, and potential biomarkers of response to therapy. Neurology. 2023;101(16):700-713. doi:10.1212/WNL.0000000000207622 37487750 PMC10585672

[11] White MK, Gordon J, Khalili K. The rapidly expanding family of human polyomaviruses: recent developments in understanding their life cycle and role in human pathology. PLoS Pathog. 2013;9(3):e1003206. doi:10.1371/journal.ppat.1003206 23516356 PMC3597531

[12] Carson KR, Newsome SD, Kim EJ, . Progressive multifocal leukoencephalopathy associated with brentuximab vedotin therapy: a report of 5 cases from the Southern Network on Adverse Reactions (SONAR) project. Cancer. 2014;120(16):2464-2471. doi:10.1002/cncr.28712 24771533 PMC4460831

[13] Pavlovic D, Patera AC, Nyberg F, Gerber M, Liu M; Progressive Multifocal Leukeoncephalopathy Consortium. Progressive multifocal leukoencephalopathy: current treatment options and future perspectives. Ther Adv Neurol Disord. 2015;8(6):255-273. doi:10.1177/1756285615602832 26600871 PMC4643867

[14] Cortese I, Reich DS, Nath A. Progressive multifocal leukoencephalopathy and the spectrum of JC virus-related disease. Nat Rev Neurol. 2021;17(1):37-51. doi:10.1038/s41582-020-00427-y 33219338 PMC7678594

[15] Balduzzi A, Lucchini G, Hirsch HH, . Polyomavirus JC-targeted T-cell therapy for progressive multiple leukoencephalopathy in a hematopoietic cell transplantation recipient. Bone Marrow Transplant. 2011;46(7):987-992. doi:10.1038/bmt.2010.221 20921942

[16] Muftuoglu M, Olson A, Marin D, . Allogeneic BK virus-specific T cells for progressive multifocal leukoencephalopathy. N Engl J Med. 2018;379(15):1443-1451. doi:10.1056/NEJMoa1801540 30304652 PMC6283403

[17] Frisque RJ, Bream GL, Cannella MT. Human polyomavirus JC virus genome. J Virol. 1984;51(2):458-469. doi:10.1128/jvi.51.2.458-469.1984 6086957 PMC254460

[18] Cortese I, Beck ES, Al-Louzi O, . BK virus-specific T cells for immunotherapy of progressive multifocal leukoencephalopathy: an open-label, single-cohort pilot study. Lancet Neurol. 2021;20(8):639-652. doi:10.1016/S1474-4422(21)00174-5 34302788 PMC8395368

[19] Priesner C, Esser R, Tischer S, . Comparative analysis of clinical-scale IFN-γ-positive T-cell enrichment using partially and fully integrated platforms. Front Immunol. 2016;7:393. doi:10.3389/fimmu.2016.00393 27746781 PMC5044705

[20] Berger JR, Aksamit AJ, Clifford DB, . PML diagnostic criteria: consensus statement from the AAN neuroinfectious disease section. Neurology. 2013;80(15):1430-1438. doi:10.1212/WNL.0b013e31828c2fa1 23568998 PMC3662270

[21] Teunissen CE, Dijkstra C, Polman C. Biological markers in CSF and blood for axonal degeneration in multiple sclerosis. Lancet Neurol. 2005;4(1):32-41. doi:10.1016/S1474-4422(04)00964-0 15620855

[22] Brettschneider J, Petzold A, Süssmuth SD, Ludolph AC, Tumani H. Axonal damage markers in cerebrospinal fluid are increased in ALS. Neurology. 2006;66(6):852-856. doi:10.1212/01.wnl.0000203120.85850.54 16567701

[23] Jiang R, Song Z, Liu L, . Survival and prognostic factors of progressive multifocal leukoencephalopathy in people living with HIV in modern ART era. Front Cell Infect Microbiol. 2023;13:1208155. doi:10.3389/fcimb.2023.1208155 38029233 PMC10663249

[24] Boumaza X, Bonneau B, Roos-Weil D, ; Immunotherapy for PML Study Group. Progressive multifocal leukoencephalopathy treated by immune checkpoint inhibitors. Ann Neurol. 2023;93(2):257-270. doi:10.1002/ana.26512 36151879 PMC10092874

[25] Leen AM, Bollard CM, Mendizabal AM, . Multicenter study of banked third-party virus-specific T cells to treat severe viral infections after hematopoietic stem cell transplantation. Blood. 2013;121(26):5113-5123. doi:10.1182/blood-2013-02-486324 23610374 PMC3695359

[26] Bleakley M, Sehgal A, Seropian S, . Naive T-cell depletion to prevent chronic graft-vs-host disease. J Clin Oncol. 2022;40(11):1174-1185. doi:10.1200/JCO.21.01755 35007144 PMC8987226

[27] Teschner D, Distler E, Wehler D, . Depletion of naive T cells using clinical grade magnetic CD45RA beads: a new approach for GVHD prophylaxis. Bone Marrow Transplant. 2014;49(1):138-144. doi:10.1038/bmt.2013.114 23933765

[28] Koralnik IJ. Overview of the cellular immunity against JC virus in progressive multifocal leukoencephalopathy. J Neurovirol. 2002;8(suppl 2):59-65. doi:10.1080/13550280290167894 12491153

