# Supplementary material for: Directly Isolated Allogeneic Virus–Specific T Cells in Progressive Multifocal Leukoencephalopathy
Source: JAMA Neurol. 2024 Oct 7;81(11):1187–98. doi: 10.1001/jamaneurol.2024.3324 (PMC11459361; doi:10.1001/jamaneurol.2024.3324)
Supplement: Supplement 1. — eMethods. eResults. eFigure 1. Overview of Donor-Selection and the Manufacturing Process of DIAVIS T Cells eFigure 2. Comparative Analysis for Cell Manufacturing Processes and Product Quality Based on Donor Source eFigure 3. Comparative Analysis of Responders and Nonresponders eFigure 4. Individual Immunomonitoring in Patients Pretreatment and 6 Weeks Posttreatment With DIAVIS T Cells eFigure 5. Correlation Between Cellular Composition, Purity, and Cell Dose in Responders (R) vs Nonresponders (NR) eFigure 6. Comparison of Survival Rate Against Historical Control of Immune Checkpoint Inhibition eFigure 7. mRS Development of Nonresponders During the Observation Period eFigure 8. Monitoring and Classifying Lesion on MRI eTable 1. Additional Characteristics of the Patients Treated With DIAVIS T Cells eTable 2. Detailed Characteristics of Best Supportive Treatment Controls eTable 3. Detailed Characteristics of Best Supportive Treatment Controls Published Within the Literature eReferences [file jamaneurol-e243324-s001.pdf]

Supplementary Online Content

Möhn N, Grote-Levi L, Wattjes MP, et al. Directly isolated allogeneic virus–specific T cells in progressive multifocal leukoencephalopathy. *JAMA Neurol*. Published online October 7, 2024. doi:10.1001/jamaneurol.2024.3324

eMethods.

eResults.

eFigure 1. Overview of Donor-Selection and the Manufacturing Process of DIAVIS T Cells

eFigure 2. Comparative Analysis for Cell Manufacturing Processes and Product Quality Based on Donor Source

eFigure 3. Comparative Analysis of Responders and Nonresponders

eFigure 4. Individual Immunomonitoring in Patients Pretreatment and 6 Weeks Posttreatment With DIAVIS T Cells

eFigure 5. Correlation Between Cellular Composition, Purity, and Cell Dose in Responders (R) vs Nonresponders (NR)

eFigure 6. Comparison of Survival Rate Against Historical Control of Immune Checkpoint Inhibition

eFigure 7. mRS Development of Nonresponders During the Observation Period

eFigure 8. Monitoring and Classifying Lesion on MRI

eTable 1. Additional Characteristics of the Patients Treated With DIAVIS T Cells

eTable 2. Detailed Characteristics of Best Supportive Treatment Controls

eTable 3. Detailed Characteristics of Best Supportive Treatment Controls Published Within the Literature

eReferences

This supplementary material has been provided by the authors to give readers additional information about their work.

© 2024 Möhn N et al. *JAMA Neurology.*

eMETHODS

PATIENT COLLECTIVE

Of the 28 patients, 26 had confirmed PML based on the detection of JC polyomavirus (JCV) via polymerase chain reaction (PCR) in their cerebrospinal fluid (CSF). For the remaining two patients, JCV was identified in a brain biopsy. Exclusion criteria for DIAVIS T-cell treatment included PML patients who had been treated with natalizumab (one patient) and those showing no signs of disease progression (three patients) (Figure 1). Clinical disease progression was defined as new symptom onset or an increase in the intensity of existing symptoms with an impact on the activities of everyday life. Patients with natalizumab-induced PML were excluded, because of the natalizumab mode of action which prevents virus-specific T cells (VSTs) from crossing the blood-brain barrier and neutralize the virus. During the same period, there were no PML patients with progressive neurological symptoms at Hannover Medical School who were not treated.

Initial outcome of two of the 28 patients were previously described[1], but this report incorporates their extended data.

MONITORING OF HPV-SPECIFIC T-CELL FREQUENCIES

Frequencies of HPV specific T-cells (JCV specific and BK polyomavirus (BKV) specific) in peripheral blood were determined by Enzyme Linked Immunospot (ELISpot) assay as described before.[2] In brief, after overnight resting in RPMI Medium (Lonza) supplemented with 10% human AB serum (c.c.pro), isolated PBMCs were stimulated with PepTivator BKV VP1, BKV LT, JCV VP1 or JCV LT (Miltenyi Biotec) at a final concentration of 1 µg/mL of each peptide overnight. Unstimulated PBMCs served as negative control, while PBMCs stimulated with 1 µg/mL staphylococcal enterotoxin B (SEB, MilliporeSigma) served as positive control. IFN-γ secretion was detected using an AID iSpot Reader System and AID ELISpot software version 8.0 (Autoimmun Diagnostika). Spots were were counted and expressed as the number of spots per well (spw). Values obtained from negative control were subtracted. Cut-off for positive response was set at ≥2 spw, with values of 2 spw considered positive in case of reactivity towards both antigens (VP1 and LT).

DONOR SELECTION AND PRE-TESTING (eFigure 1 and eFigure 2)

DIAVIS T-cells were obtained directly from healthy, partially HLA-matched donors, pretested for their frequencies of BK polyomavirus (BKV) specific T-cells. Appropriate third-party donors were either first-degree relatives (family donors, FD), or unrelated individuals (unrelated donors, UD) selected from the pre-screened alloCELL registry

© 2024 Möhn N et al. *JAMA Neurology.*

(Hannover, Germany) based on the level of HLA match. Besides the HLA match, the frequencies of BKV-specific T-cells were decisive in the T-cell donor selection. A prior BKV antibody test was not performed on the donors, as antibody detection does not automatically indicate sufficient presence of virus-specific T cells (antibody prevalence up to 90 %[3] versus up to 50% prevalence of BKV specific VSTs[4]). As FD inherently show the potential to have an HLA match of at least 5/10 and are usually personally involved, their involvement can possibly speed up the donor selection process. The IFN-γ cytokine secretion assay (CSA, Miltenyi Biotec) was used to determine the frequency of BKV specific T-cells, and was performed as previously described.[5] In brief, after overnight resting in TexMACS Medium (Miltenyi Biotec), isolated PBMCs were cultured at a concentration of $1x10^7$ cells/mL and stimulated with a combination of PepTivator BKV VP1 and BKV LT peptide pool (Miltenyi Biotec) at a final concentration of 1 µg/mL of each peptide. Unstimulated PBMCs served as negative control. Using anti-IFN-γ phycoerythrin (PE) antibodies and paramagnetic anti-PE microbeads, activated IFN-γ-secreting T-cells were specifically isolated by a magnetic enrichment. Pre- and post-enrichment cell fractions were stained for flow cytometric analysis using 7-amino-actinomycin D (7-AAD, BD Biosciences, Heidelberg, Germany), anti-CD45 APC-H7, anti-CD3 Fluorescein Isothiocyanate (FITC), anti-CD8 allophycocyanine (APC) and anti-CD4 Alexa Fluor 700 (AF700) antibodies (all from BD Biosciences). At least 10,000 events were acquired in the viable (7-AAD negative) $CD45^+$ leukocyte gate (FACSCanto10c, BD Biosciences, Heidelberg, Germany).

CLINICAL GRADE MANUFACTURING OF DIAVIS T-CELLS (eFigure 1 and 2)

Clinical-grade BKV specific DIAVIS T-cells were produced from healthy donors under GMP conditions. At the time of manufacturing (2020-2022), JCV-derived PepTivators were not commercially available in GMP quality. Therefore, we used BKV-derived PepTivators in GMP quality. As both viruses have a high sequence homology and match in the key antigens (including VP1 and LT), the use of BKV specific T-cells has the potential to also induce an anti-JCV response by cross-reactivity. Clinical-grade BKV specific T-cells were isolated from leukapheresis products (LP) of pretested donors, using the CliniMACS Prodigy device (Miltenyi Biotec) and the CliniMACS CCS IFN-gamma (Miltenyi Biotec) according to manufacturer's instructions and as previously described.[6, 7] In brief, $1x10^9$ nucleated cells from the LP products (counted using the coulter ACTdiff, Beckman Coulter, Krefeld, Germany) were used for an automated enrichment process: Cells were stimulated with a combination of MACS GMP PepTivator BKV VP1 and BKV LT (1 µg/ml per peptide, Miltenyi Biotec) for 4 h at 37 °C and 5% CO_2), followed by labelling of white blood cells with the CliniMACS CCS Catchmatrix Reagent during the cytokine secretion phase, allowing immunomagnetic separation of IFN-γ-producing cells by anti-IFN-γ

© 2024 Möhn N et al. *JAMA Neurology.*

antibodies, conjugated to super-paramagnetic particles (CliniMACS IFN-γ Enrichment Reagent, Miltenyi Biotec). A quality control sample (pre drug substance, preDS) prior to magnetic enrichment was taken from the quality control bag.

Quality control of LP, preDS and the enriched T-cell product (drug substance, DS) was performed as previously described.[8] Using Trucount Absolute Counting Tubes (BD Biosciences), anti-CD45 APC-H7, anti-CD3 FITC and 7-AAD (all BD Biosciences), total $CD45^+$ leukocytes, viability, and frequencies, as well as total viable $CD3^+$ T-cell numbers were determined by flow cytometry (BD FACSCanto 10c). At least 10,000-50,000 events were acquired in the Trucount beads gate. Using anti-CD45 APC-H7, anti-CD3 FITC, anti-CD4 AF700, anti-CD8 APC, anti-CD14 BV510, anti-CD19 BV510, anti-IFN-γ PE and 7-AAD (all BD Biosciences), cellular composition and purities were determined (BD FACSCanto 10c). At least 10,000-50,000 events were acquired in the $CD45^+$ leukocyte gate. A suitable T-cell product could be manufactured for all patients, there was no unsuccessful collection.

HISTORICAL CONTROLS

To establish a historical control group, a PubMed literature search was conducted, including case reports and series from 2014 to 2022 using "PML" and "progressive multifocal leukoencephalopathy" as search terms. Apart from a case series from 2014 by Lee et al including two PML-patients[9], only single patient reports were integrated. Cases lacking diagnostic or survival duration data were excluded. Only PML patients with underlying lymphoproliferative, autoimmune diseases or unclear lymphopenia were analyzed. Additionally, PML patients from this group who received experimental treatment with checkpoint inhibitors (anti-PD-1 antibodies) were analyzed separately. Cases from the literature search were complemented by additional PML cases treated at the Department of Neurology, University Hospital of Cologne, and Hannover Medical School from 2006 to 2022. A total of nine of these patients were diagnosed with PML between March 2020 and February 2022. In all of these cases, the patients or the respective legal representatives decided against experimental PML therapy. The four cases that were initially screened for T-cell therapy but were ultimately not treated (Figure 1) were not included in the 26 unpublished cases because they were either treated with natalizumab (n=1) or did not show progressive neurological symptoms (n=3). In two of these patients who did not experience disease progression, PML was attributed to AIDS, and treatment with HIV therapy resulted in the stabilization of PML. In the third patient, PML was stabilized after treatment with pembrolizumab. In this cohort of published and unpublished cases, only patients not treated with DIAVIS T-cells or anti-PD-1 antibodies were included. This historical control was thus defined as cohort of patients receiving best supportive treatment (BST-control). The BST-control group

© 2024 Möhn N et al. *JAMA Neurology.*

consisted of 113 cases, comprising 59 patients with lymphoproliferative conditions, 34 with systemic autoimmune diseases, 14 with lymphopenia, and six with mixed conditions including lymphoproliferative disease combined with autoimmune hemolytic anemia, HIV, solid tumor, or organ transplant (eTable 2 and 3).[9-95] AIDS-related PML cases were omitted. This historical control group closely matched our DIAVIS T-cell-treated cohort in terms of PML causes. Due to the similar distribution of the underlying diseases and the identical median age of 60 years in both cohorts, it can be assumed that the BST-control group and the cohort of DIAVIS-T-cell treated patients are meaningfully similar in terms of PML prognosis. The detailed clinical characteristics can be obtained from eTable 2 and 3.

A second historical control group of patients experimentally treated with checkpoint inhibitors (anti-PD-1 antibodies), defined as ICI-controls, was used to compare survival times with patients treated with DIAVIS T-cells. This patient group was recently published by Boumaza et al., who conducted a multicenter survey compiling retrospective data from 79 PML patients.[96] One year follow-up data were analyzed to determine clinical outcomes and safety profile. To form the second control group, 12 patients with AIDS as underlying disease were excluded from the cohort. Hence, the second historical control group comprised 67 PML patients with underlying conditions including lymphoproliferative diseases (38 patients), primary immunodeficiency (14 patients), autoimmune diseases (eight patients), neoplasms (five patients), and kidney transplantation (two patients). Of the 67 patients, the majority were men (66%) and the median age amounted to 65 years. The median mRS at baseline was 3. Due to the similar distribution of underlying diseases and the similar age of the patients in this control cohort, it can be assumed that the prognosis of the affected patients is comparable to that of the T-cell-treated ones. It should be emphasized that the cohort at baseline was more mildly affected (mRS 3) than the cohort of DIAVIS T-cell-treated patients (mRS 4).

© 2024 Möhn N et al. *JAMA Neurology.*

eRESULTS

PATIENT SELECTION PROCEDURE (Figure 1)

Of the 38 patients evaluated for potential PML treatment, four were excluded: one was ineligible due to PML induced by natalizumab, and the other three were ruled out as they exhibited PML but lacked clinical and radiological signs of disease progression. These three were clinically monitored for a minimum of six months and underwent at least three brain MRI scans in other facilities, consistently showing clinical and radiological stability. Of the 34 patients left, all were assessed for the presence of endogenous BKV and JCV specific T-cells in their peripheral blood. In 24 of these patients, no virus-specific T-cells were detected, leading to their selection for DIAVIS T-cell therapy. It must be emphasised that all patients, regardless of the severity of their PML-related symptoms, were selected for treatment and that there has been no selection bias for less severe disease. Virus-specific T cells were identified in nine additional patients. Three of these patients, having no contraindications for immune checkpoint inhibitors (ICI), were treated with pembrolizumab. The other six patients had systemic diseases, making them ineligible for ICI treatment. Consequently, they were considered for DIAVIS T-cell therapy as well. For the remaining two patients, the presence of virus-specific T-cells could not be definitively confirmed due to technical problems. One of these patients had systemic autoimmune disease as a contraindication for immune checkpoint inhibitors, and the other was previously treated with pembrolizumab that did respond to treatment. Therefore, both were considered for DIAVIS T-cell therapy. In total, a donor search for DIAVIS T-cell therapy was initiated in 31 cases. Of these 31 patients, three did not live long enough to receive the treatment: one died due to sepsis 23 days after T-cell analysis, another experienced rapid deterioration and passed away after 15 days, and the third, facing swift disease progression, decided against the experimental therapy and died 29 days later. At the end, a total of 28 patients diagnosed with PML, and experiencing disease progression, underwent treatment with DIAVIS T-cells.

DONOR PRE-TESTING (eFigure 1)

Several potential (partially) HLA-matched donors per patient were screened with respect to BKV-specific T-cell frequencies. Overall, donor pre-testing (determination of BKV-specific T-cell frequencies) was performed for 4 potential donors per patient (range 1-7). The pre-testing results for donors that were selected for manufacturing are shown in eFigure 1. In selected donors, mean frequencies of BKV specific T-cells ($CD3^+$/IFN-γ^+) were 0.10% (range 0.01-0.66%) (eFigure 1B), which were increased to 28.61% (range 11.28-80.57%) after magnetic enrichment (eFigure 1C). Despite the fact that frequencies of T-cells specific for BKV are markedly lower when

© 2024 Möhn N et al. *JAMA Neurology.*

compared to e.g. herpesviruses such as Cytomegalovirus and Epstein-Barr Virus[4], (partially) HLA-matched donors with sufficient frequencies of BKV-specific T-cells suitable for manufacturing of DIAVIS T-cells could be identified for each patient.

CHARACTERISTICS OF THE PATIENT COHORT THAT RECEIVED DIAVIS T-CELLS (eTable 1)

Out of the 17 patients with lymphoproliferative disorders, 11 had received B-cell depleting therapies. Additionally, eight had received either autologous (n=4) or allogeneic (n=4) stem cell transplantation to treat their primary disease. Of the five patients with underlying autoimmune diseases, four had previously received immunosuppressive therapy (prednisolone (n=3), methotrexate and rituximab (n=1)). In a single patient, the pre-existing sarcoidosis was first diagnosed due to PML. In two patients, immunosuppression was discontinued at the time of PML diagnosis. In the two remaining patients, prednisolone was maintained at a low dose, with one patient also discontinuing additional medication with methotrexate and rituximab at the time of PML diagnosis. Of the 28 patients treated, 22 had not previously undergone any experimental immunomodulatory or antiviral therapy. Elsewhere, three out of the remaining six patients had received Pembrolizumab as an experimental treatment; however, this approach was ineffective and the patients experienced disease progression both clinically and on imaging. Another patient had been treated with interleukin 2; while she initially showed stabilization of the PML, her condition worsened over time. Two patients with AIDS had been previously treated with antiretroviral therapy, starting six month and seven years with change of medication twelve month before. In both cases, PML occurred despite sufficient antiretroviral therapy, with $CD4^+$ T-cell counts having increased to 117/µl and 556/µl, respectively. HIV viral load was below the quantification limit (< 30 copies/ml) and not detectable. However, in both cases no BKV or JCV specific T-cells were identified in the patients' blood.

OUTCOMES

Clinical data included outcomes of the modified Rankin Scale (mRS), Montreal Cognitive Assessment (MoCA), and routine patient examinations. CSF analyses provided insights into the JCV viral load, as well as neuronal damage markers such as phosphorylated neurofilament heavy chain (pNfH) and total Tau protein (tTau). pNfH has a high specificity for axonal damage and has proven its value, for example, in the diagnosis of amyotrophic lateral sclerosis (ALS).[97, 98] In the context of PML, pNfH was selected for measurement as it signifies axonal damage in the brain, a distinctive feature of PML.[99] Analysis of pNFH was performed at the University of Ulm using commercially available ELISA kit (Biovendor, Heidelberg, Germany) according to the manufacturers' instructions. For Tau analysis, Elecsys CSF immunoassay was used according to the manufacturers' instructions.

© 2024 Möhn N et al. *JAMA Neurology.*

Multisequence brain MRI scans including T2-FLAIR, T2-weighted turbo/fast spin echo, DWI, and contrast-enhanced T1-weighted, were used to monitor inflammation in the brain. Except for one patient who required dialysis due to renal insufficiency and therefore did not receive gadolinium, all patients were administered gadolinium before MRI-imaging. MR images were rated regarding lesion location, lesion distribution (unilobar, multilobar, widespread) and signs of inflammation with special regard to contrast-enhancement (eFigure 8). On follow-up lesion evolution in terms of size and changes in contrast-enhancement were rated visually and changes in terms of size validated by measurements in two different anatomic planes. At the three-month follow-up, the brain MRI of one patient was conducted but could not be evaluated. Additionally, MRI scans were unavailable for one patient at six weeks, two patients at three months, and another two patients at six months post-therapy initiation.

BKV/JCV specific T-cells were measured in EDTA blood samples. Additionally, potential side effects were monitored. Vital signs were closely monitored to detect any immediate reactions following DIAVIS T-cell infusion. Indications of graft-versus-host disease (GvHD) were followed through comprehensive medical history, physical evaluations, and laboratory testing with a focus on levels of liver transaminases.

© 2024 Möhn N et al. *JAMA Neurology.*

SUPPLEMENTARY TABLES AND FIGURES

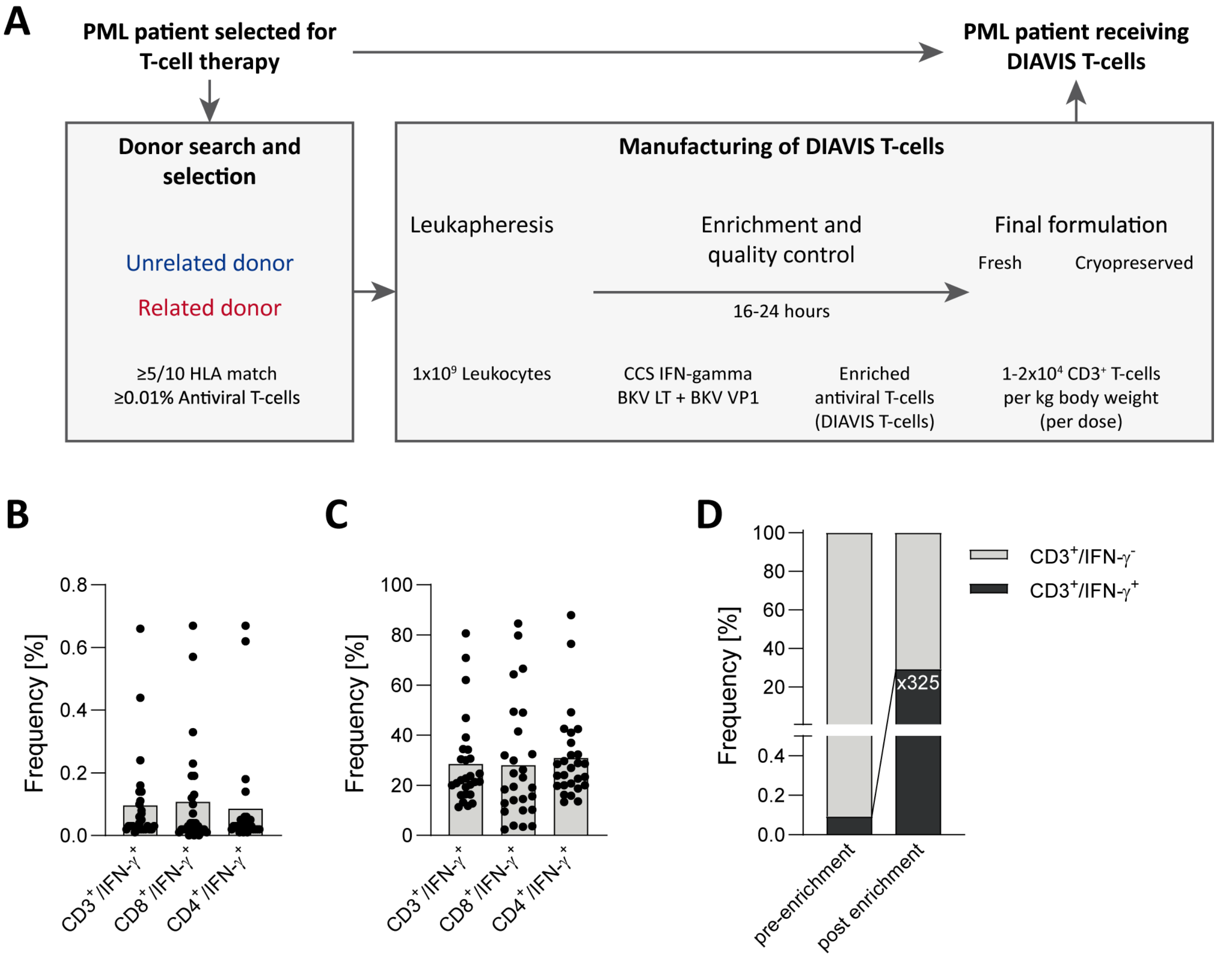

eFigure 1: Overview of donor-selection and the manufacturing process of DIAVIS T-cells

(A) At the beginning of the process, it is essential to find a suitable third-party donor who is at least partially HLA-matched and has a high frequency of BKV-specific T-cells. The next step involves isolating clinical-grade BKV specific T-cells from the donor's leukapheresis product. This isolation is done using the CliniMACS® Prodigy device and the CliniMACS® CCS system (IFN-gamma), both from Miltenyi Biotec. After an overnight rest period, $1x10^9$ white blood cells are stimulated with the overlapping peptide pools covering the sequences of the BKV LT and BKV VP1 proteins. The activated IFN-γ-secreting BKV specific T-cells are then selectively enriched through a magnetic process utilizing specific antibodies and paramagnetic microbeads. These enriched cells are termed DIAVIS T-cells. The final therapeutic product is targeted to have a cell dose of $1\text{-}2x10^4$ $CD3^+$ T-cells per kilogram of body weight. One dose of these cells is administered fresh, while the remaining doses are cryopreserved for future use. (B, C) Donor pre-testing data show the frequencies of BKV specific T-cells (IFN-γ^+) amongst $CD3^+$, $CD8^+$ and $CD4^+$ T-cells pre-enrichment (B) as well as after magnetic enrichment (C) determined by IFN-γ Cytokine Secretion Assay. (D) Enrichment of BKV specific T-cells ($CD3^+$/IFN-γ^+) during clinical-grade manufacturing.

© 2024 Möhn N et al. *JAMA Neurology.*

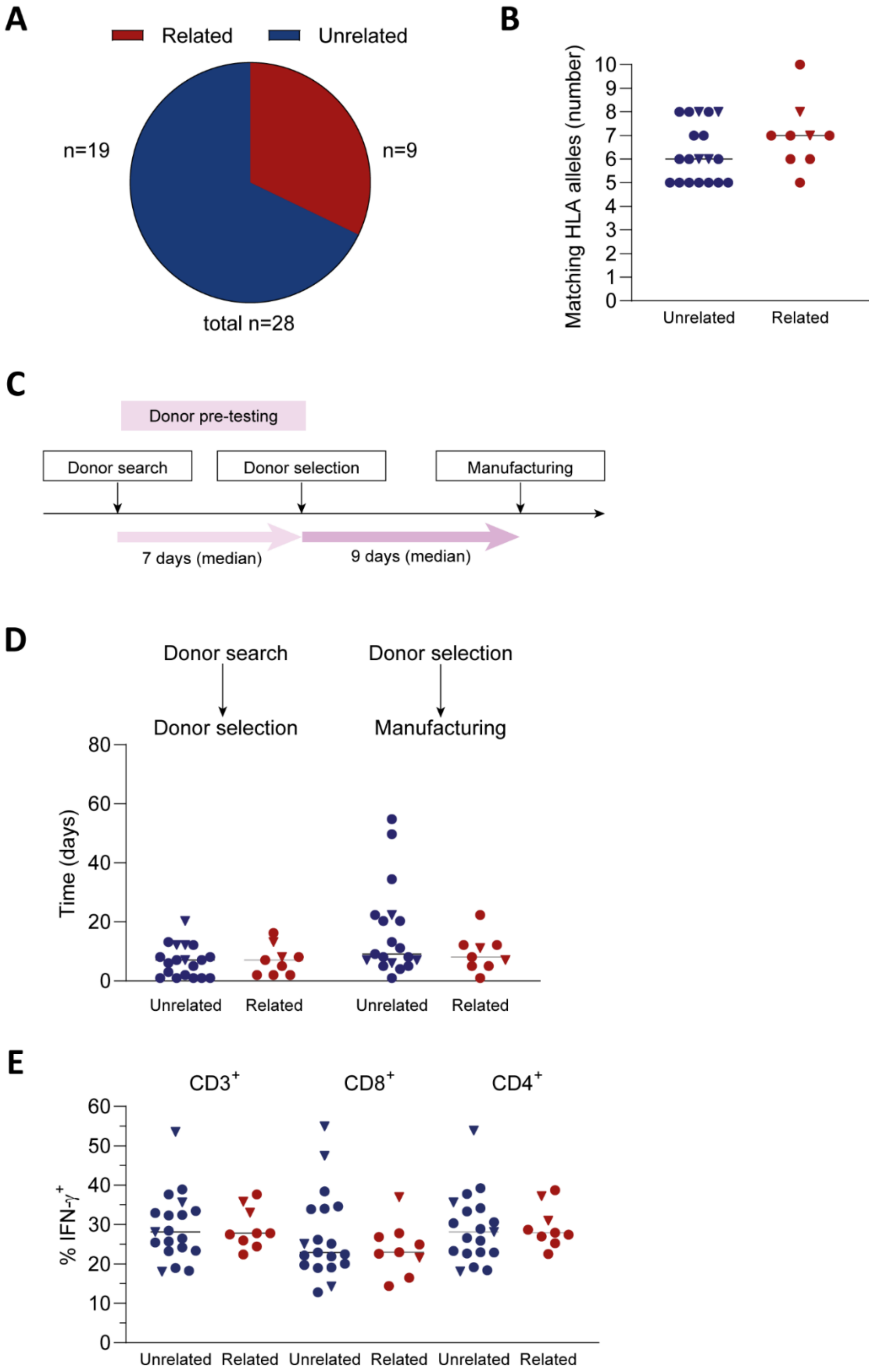

eFigure 2: Comparative analysis for cell manufacturing processes and product quality based on donor source
The data shows the proportion of unrelated and related donors (A) as well as the proportion of matching HLA-alleles in these donors (B). Sketch depicting the median timelines from donor search to donor selection, and from donor selection to the manufacturing of virus-specific T-cells (C), along with a comparison of the individual duration of process steps between unrelated and related donors (D). Frequencies of IFN-γ^{+} cells within the CD3+, CD8+, and CD4+ cell populations in final cell product, comparing samples from unrelated and related donors (E). Triangle defining non-responder, dots defining responder parameters.

© 2024 Möhn N et al. *JAMA Neurology.*

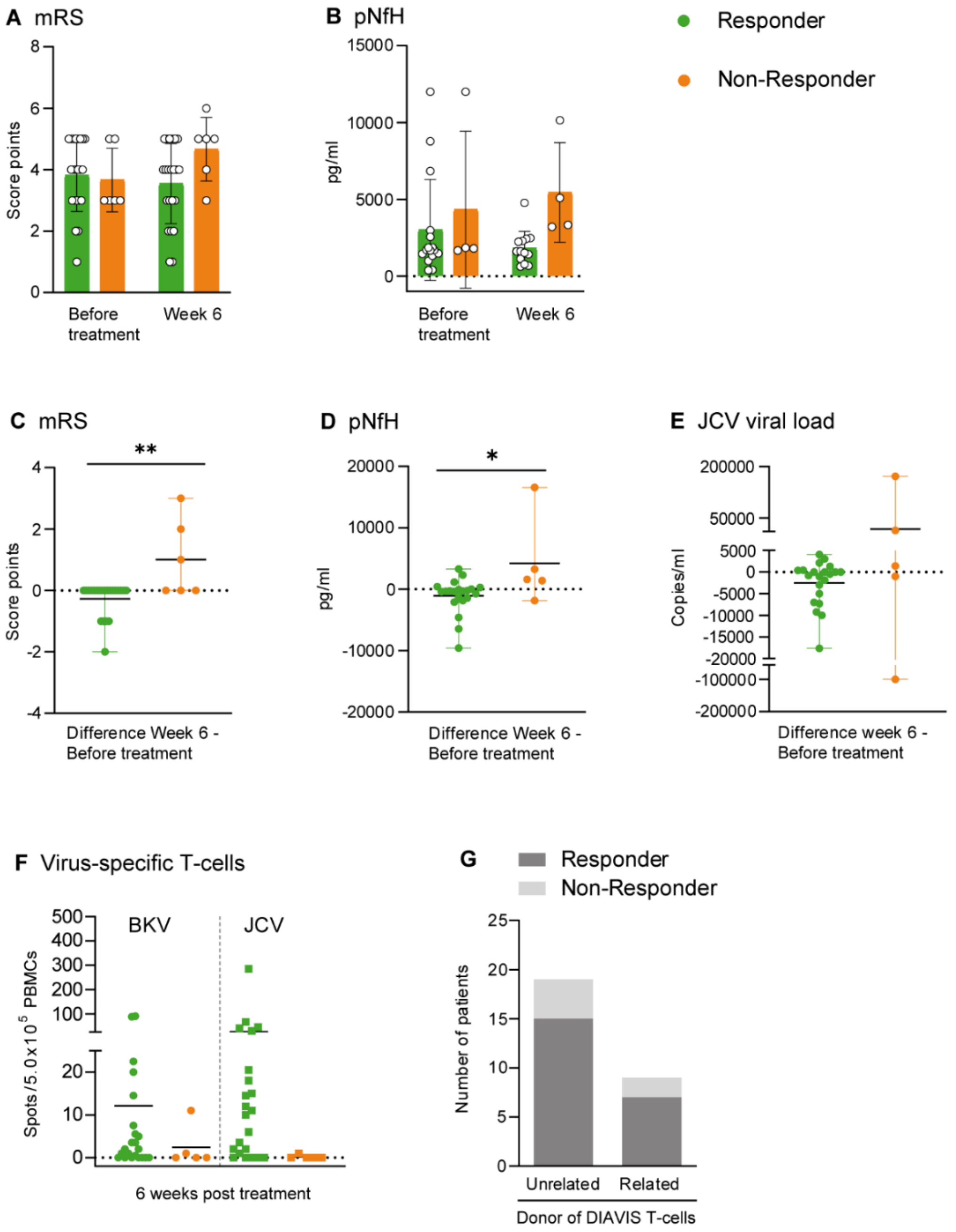

eFigure 3: Comparative analysis of responders and non-responders

Modified Rankin Scale (mRS) scores in treatment responders and non-responders, comparing values before and after 6 weeks of treatment (A), and the changes observed in mRS scores from pre-treatment to week 6 in both groups (C). Cerebrospinal fluid (CSF)-values of phosphorylated neurofilament heavy chain (pNfH) in treatment responders and non-responders, comparing values before and after 6 weeks of treatment (B), and the changes observed in pNfH from pre-treatment to week 6 in both groups (D). CSF-values of JC polyomavirus (JCV) viral load in treatment responders and non-responders comparing the changes observed in viral load from pre-treatment to week 6 in both groups (E). Total proportion of detected virus-specific T-cells (VST) in the blood of responders and non-responders 6 weeks after treatment (F). Fraction of responders and non-responders in unrelated and related T-cell donors (G). A and B show mean with standard deviation, while C, D and E show mean with range. Week 6 = median day 43 (IQR 40-48, minimum-maximum 13-65).

© 2024 Möhn N et al. *JAMA Neurology.*

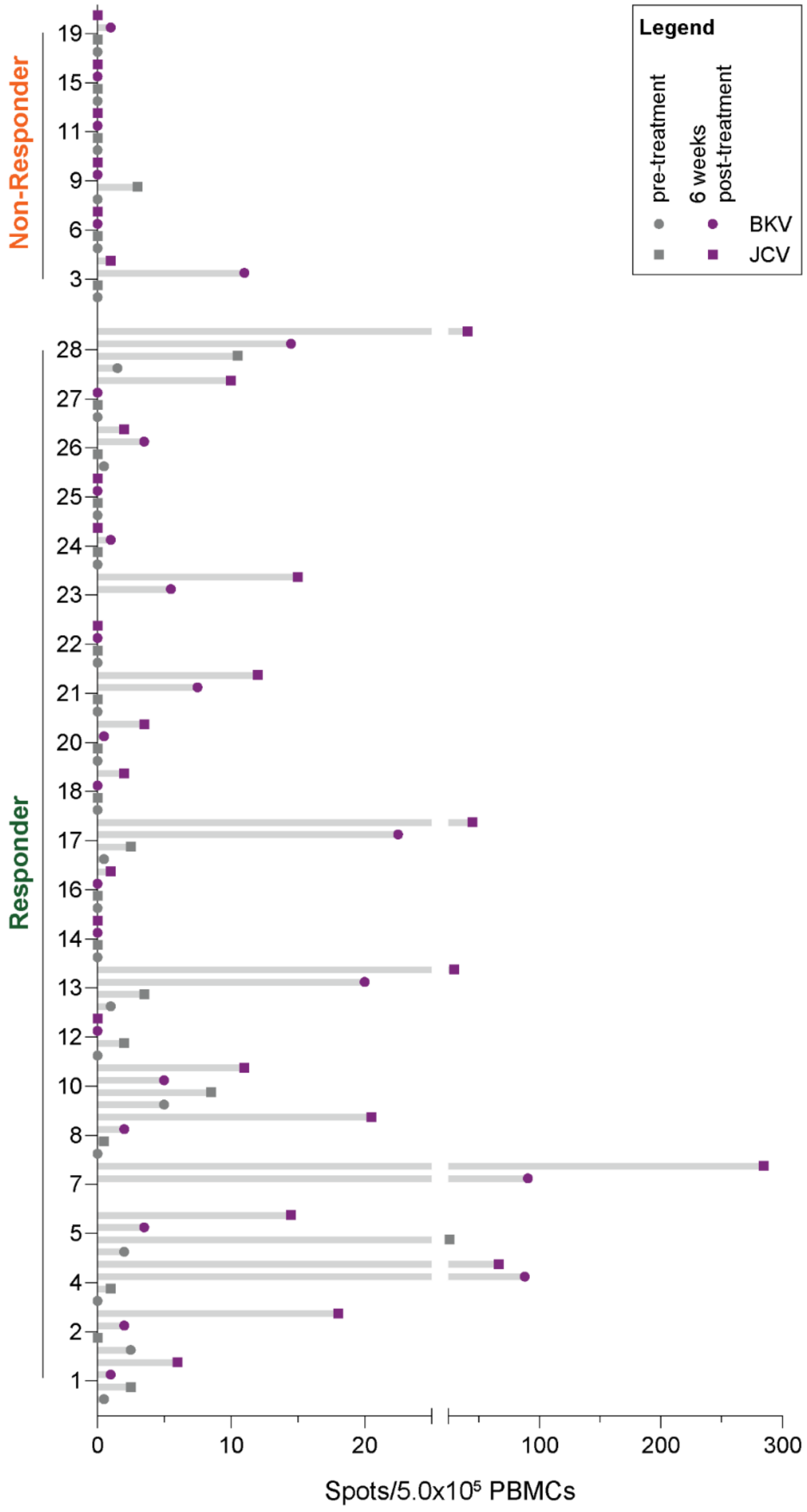

eFigure 4: Individual immunomonitoring in patients pre-treatment and six weeks post treatment with DIAVIS T-cells

The graph shows the frequencies of BKV (circles) and JCV specific (squares) T-cells in peripheral blood of patients pre-treatment (grey) and six weeks post treatment with DIAVIS T-cells (purple) determined by IFN-γ Enzyme Linked Immunospot (ELISpot) assay. Data are shown as spots per well (spw) per $5.0x10^5$ PBMCs (values from unstimulated control are subtracted).

© 2024 Möhn N et al. *JAMA Neurology.*

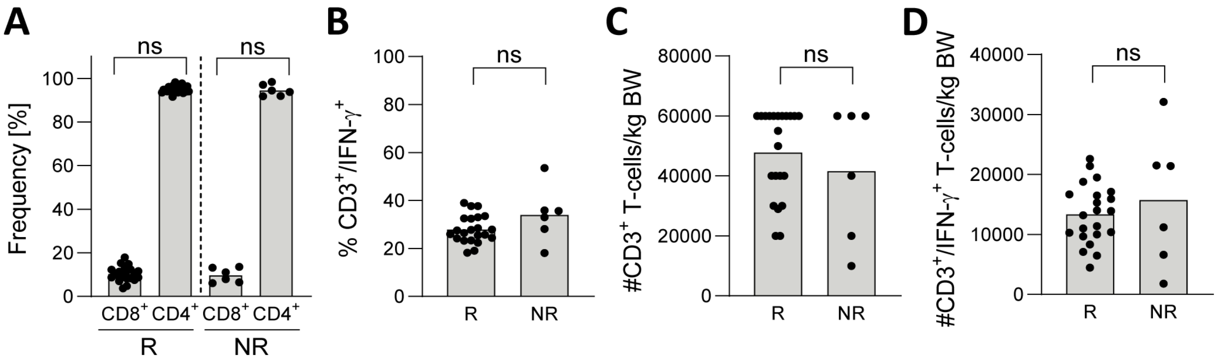

eFigure 5: Correlation between cellular composition, purity and cell dose in responders (R) versus non-responders (NR)

The data shows the frequencies of CD8⁺ and CD4⁺ T-cells (A) and the purity (%CD3+/IFN-g+) of the DIAVIS T-cell product, as well as the total applied CD3⁺ T-cell dose/kg BW (C) and the total applied BKV specific T-cell dose (CD3⁺/IFN-γ⁺). ns: not significant (unpaired t-test).

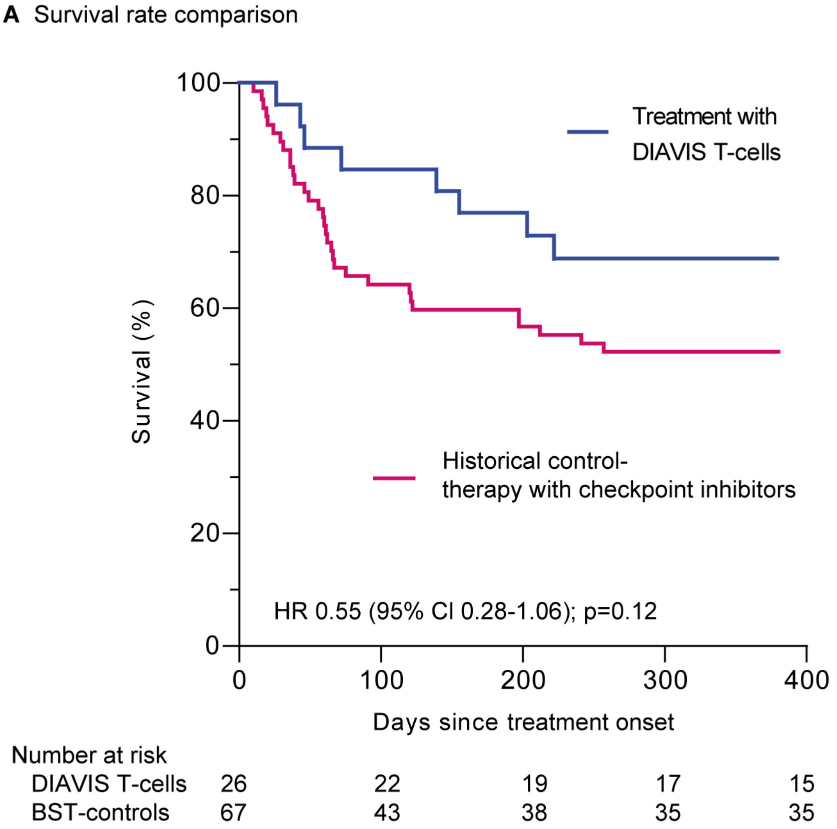

eFigure 6: Comparison of survival rate against historical control of immune checkpoint inhibition

A comparison of the survival curves of patients treated with directly isolated allogeneic virus-specific (DIAVIS) T-cells with historical controls that had undergone treatment with immune checkpoint inhibitors (ICI-controls).

© 2024 Möhn N et al. *JAMA Neurology.*

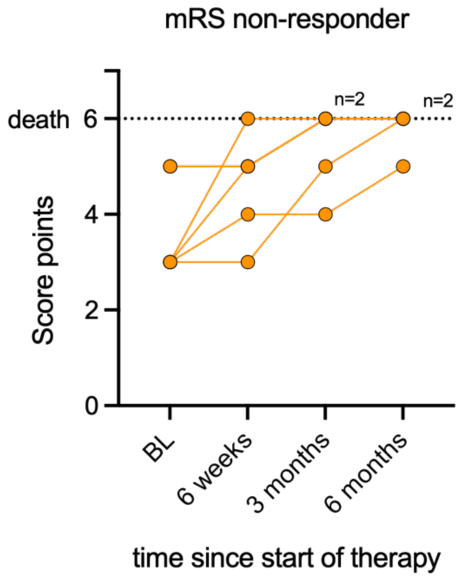

eFigure 7: mRS development of non-responders during the observation period
Illustration of the change in the modified Ranking Scale (mRS) score of non-responders over a period of 6 months. An mRS score of 6 is equivalent to the death of the patient.

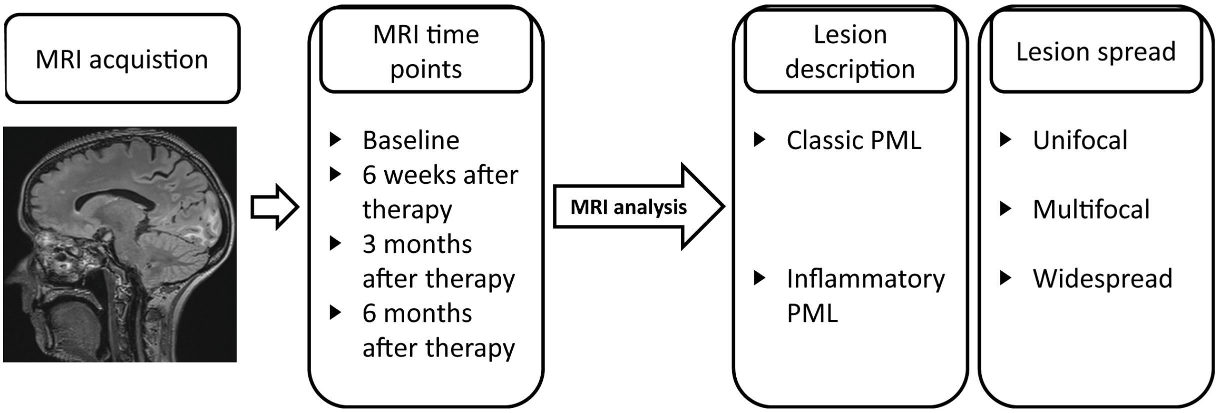

eFigure 8: Monitoring and classifying lesion on MRI
This flowchart outlines the MRI workflow used to monitor lesions in patients. The MRI scans are conducted at specific time points: Baseline (before therapy), 6 weeks after therapy initiation, 3 months after therapy, and 6 months after therapy. Based on MRI analysis, lesions are categorized into two types: Classic Progressive Multifocal Leukoencephalopathy (PML) and Inflammatory PML, depending on the presence of inflammation. The extent of lesion spread is then classified as Unifocal, Multifocal or Widespread. This structured approach enables systematic monitoring of lesion progression and response to therapy.

© 2024 Möhn N et al. *JAMA Neurology.*

eTable 1. Additional characteristics of the patients treated with DIAVIS T-cells

Patient no.	Previous CD20 depletion	Previous stem cell therapy	Confirmation of PML diagnosis via	Previous experimental therapy of PML	Observed disease progression (days)
1	Yes	Autologous	CSF	No	93
2	No	No	CSF	No	50
3	Yes	allogeneic	CSF	No	44
4	No	No	CSF	No	50
5	No	No	Brain biopsy	No	105
6	No	No	CSF	No	157
7	No	No	Brain biopsy	Pembrolizumab	67
8	No	No	Brain biopsy	No	207
9	Yes	No	CSF	No	106
10	No	Allogeneic	Brain biopsy	No	42
11	No	No	CSF	No	123
12	No	no	CSF	No	71
13	No	No	Brain biopsy	No	21
14	No	No	CSF	Pembrolizumab	130
15	Yes	Autologous	Brain biopsy	No	157
16	Yes	No	Brain biopsy	Pembrolizumab	174

© 2024 Möhn N et al. *JAMA Neurology.*

17	No	Allogeneic	CSF	No	91
18	No	No	CSF	No	89
19	Yes	No	CSF	No	39
20	No	Autologous	CSF	No	49
21	No	No	Brain biopsy	No	163
22	Yes	Allogeneic	CSF	No	56
23	Yes	No	CSF	No	90
24	Yes	Autologous	CSF	IL-2	65
25	Yes	No	Brain biopsy	No	102
26	No	No	CSF	No	46
27	Yes	No	CSF	No	199
28	No	No	CSF	No	46

eTable 1: Additional characteristics of the patients treated with DIAVIS T-cells

Clinical characteristics of patients treated with DIAVIS T-cells are shown. CD20 depletion therapies included: Rituximab or Obinutuzumab. CSF = cerebrospinal fluid

© 2024 Möhn N et al. *JAMA Neurology.*

**eTable 2. DETAILED CHARACTERISTICS OF BEST SUPPORTIVE TREATMENT-CONTROLS**					
**Characteristics**	All patients (n=113)	Internal patients (n=26)	Patients published (n=87)	Comparison internal versus published patients Difference (CI 95%)	Comparison internal versus published patients p-value
Females/males, n	50/63	10/16	40/47	0 (0 to 0)	0.08
Age, median (IQR)	60 (47-68)	61 (52-72)	60 (45-67)	4.13 (-2.36 to 10.62)	0.21
Underlying disease					0.67
Lymphoproliferative disorders, *n*	58 (52%)	16 (61%)	42 (48%)		
Autoimmune diseases, *n*	34 (30%)	6 (23%)	28 (32%)		
Lymphopenia, *n*	14 (12%)	2 (8%)	12 (14%)		
Lymphoproliferative disease + further immunosuppressive condition, *n*	7 (6%)	2 (8%)	5 (5%)		
Survival at last follow up					
Alive, *n*	53 (47%)	14 (54%)	39 (45%)	1 (0 to 0)	0.5
Deceased, *n*	60 (53%)	12 (46%)	48 (55%)	-1 (0 to 0)	0.5
Time of death after PML diagnosis (days), median (IQR)	60 (30-134)	45 (29-88)	60 (37-147)	-26.98 (-131.6 to 77.6)	0.61

eTable 2: Detailed characteristics of best supportive treatment-controls

Clinical characteristics of patients treated with best supportive treatment are shown. IQR = interquartile range. Statistical analysis of distribution of underlying disease was performed with Fisher´s exact test.

© 2024 Möhn N et al. *JAMA Neurology.*

eTable 3. DETAILED CHARACTERISTICS OF BEST SUPPORTIVE TREATMENT-CONTROLS PUBLISHED WITHIN THE LITERATURE								
No.	Year of publication	Source of data	Age at PML diagnosis	Sex	Underlying cause of PML	Category	Survival	Last follow up/ time of death after PML diagnosis (days)
1	2018	Ishii J, Shishido-Hara Y, Kawamoto M, et al.	37	f	SLE	Autoimmune disease	Alive	690
2	2017	Ikeda J, Matsushima A, Ishii W, et al.	32	f	SLE	Autoimmune disease	Alive	730
3	2018	Ishikawa Y, Kasuya T, Ishikawa J, Fujiwara M, Kita Y.	36	m	SLE	Autoimmune disease	Alive	330
4	2019	Cheng CF, Su JJ, Chen YF, Lin YC, Huang YM, Li KJ.	27	f	SLE, rheumatoid arthritis	Autoimmune disease	Alive	365
5	2016	Trentalange A, Calcagno A, Ghisetti V, et al.	68	f	Sarcoidosis	Autoimmune disease	Alive	365
6	2019	Nishigori R, Warabi Y, Shishido-Hara Y, et al.	74	f	Rheumatoid arthritis	Autoimmune disease	Alive	170
7	2015	Dammeier N, Schubert V, Hauser TK, Bornemann A, Bischof F.	53	f	Psoriasis	Autoimmune disease	Alive	210
8	2015	Bartsch T, Rempe T, Wrede A, et al.	68	m	Psoriasis	Autoimmune disease	Alive	365
9	2016	Ellrichmann G, Behrendt V, Grunwald C, Schlottmann R, Lukas C, Gold R.	38	m	Sarcoidosis	Autoimmune disease	Alive	270
10	2019	Nosaki Y, Ohyama K, Watanabe M, et al.	65	f	Rheumatoid arthritis	Autoimmune disease	Alive	180
11	2017	Tirelli L, Rosini F, Rufa A, et al.	39	f	SLE	Autoimmune disease	Alive	730
12	2015	Kurmann R, Weisstanner C, Kardas P, et al.	56	m	common variable immunodeficiency, Idiopathic CD4+ lymphocytopenia	Lymphopenia	Alive	690
13	2017	Nambirajan A, Suri V, Kataria V, Sharma MC, Goyal V.	44	m	Idiopathic CD4+ lymphocytopenia	Lymphopenia	Alive	330
14	2020	Dato C, Elefante A, Coppola C, et al.	40	m	Idiopathic CD4+ lymphocytopenia	Lymphopenia	Alive	730
15	2018	Harel A, Horng S, Gustafson T, Ramineni A, Farber RS, Fabian M.	63	w	Idiopathic CD4+ lymphocytopenia	Lymphopenia	Alive	730
16	2016	Gupta HV, Gokden M, Ramakrishnaiah RH, Archer RL.	33	m	Idiopathic CD4+ lymphocytopenia	Lymphopenia	Alive	365
17	2020	Oza A, Rettig MP, Powell P, et al.	27	m	T-cell acute lymphoblastic leukemia	Lymphoproliferative	Alive	926
18	2019	Castro-Sánchez MV, Villagrán-García M, Romero-Imbroda J.	81	m	Low-grade NHL	Lymphoproliferative	Alive	120

© 2024 Möhn N et al. *JAMA Neurology.*

19	2019	Masuoka K, Akagawa Y, Hanashiro R, Yamaguchi M, Oota H, Kiyozuka T.	68	f	Severe aplastic anemia	Lymphoproliferative	Alive	180
20	2020	Ueno H, Kikumto M, Takebayashi Y, et al.	69	f	Multiple myeloma	Lymphoproliferative	Alive	150
21	2016	Yokokawa K, Hisahara S, Matsuura Y, et al.	62	m	Multiple myeloma	Lymphoproliferative	Alive	100
22	2015	Garrote H, de la Fuente A, Oña R, et al.	50	m	CLL	Lymphoproliferative	Alive	1140
23	2016	Sanjo N, Kina S, Shishido-Hara Y, et al.	53	m	Follicular lymphoma	Lymphoproliferative	Alive	365
24	2017	Lam W, Al-Shaibani Z, Kumar D, et al.	52	m	AML	Lymphoproliferative	Alive	365
25	2019	Pasca M, Picchioni A, Mazzeo S, et al.	44	f	Subcutaneous panniculitis-like T-cell lymphoma	Lymphoproliferative	Alive	570
26	2018	Hsiehchen D, Arasaratnam R, Raj K, Froehlich T, Anderson L.	70	f	CLL	Lymphoproliferative	Alive	14
27	2021	Nakamura R, Kitamura A, Tsukamoto T, et al.	65	m	AML	Lymphoproliferative	Alive	117
28	2020	Mosna K, Ladicka M, Drgona L, et al.	62	f	Mantle cell lymphoma	Lymphoproliferative	Alive	120
29	2018	Martinot M, Ahle G, Petrosyan I, et al.	54	m	Hodgkin lymphoma	Lymphoproliferative	Alive	150
30	2016	Kelly D, Monaghan B, McMahon E, et al.	60	m	Follicular lymphoma	Lymphoproliferative	Alive	365
31	2019	Amano E, Ozaki K, Ishibashi S, Sanjo N, Yokota T.	38	f	Autoimmune hemolytic anemia, lymphoid tissue lymphoma	Mixed condition	Alive	180
32	2019	Peña M, Presas-Rodríguez S, Ribera JM.	74	m	T-cell leukemia, autoimmune hemolytic anemia	Mixed condition	Alive	90
33	2020	Godbole MM, Barr PM.	77	m	Prostate cancer, mantel cell lymphoma	Mixed condition	Alive	305
34	2014	Skovrlj B, Rasouli J, Caridi J, Taylor WM, Galyon DD.	51	m	Morbus Waldenstrom	Lymphoproliferative	Alive	366
35	2014	Alstadhaug KB, Croughs T, Henriksen S, et al.	69	m	Idiopathic CD4+ lymphopenia	Lymphopenia	Alive	514
36	2014	Berger MD, Meisel A, Andres M, Schanz U, Schwarz U, Stussi G.	45	f	AML	Lymphoproliferative	Alive	610
37	2014	Mungunkhuyag M, Harada M, Abe T, Fujita K, Matsui N, Kaji R.	55	m	Multiple myeloma	Lymphoproliferative	Alive	244
38	2014	Yoshida H, Ohshima K, Toda J, et al.	38	f	Myelofibrosis	Lymphoproliferative	Alive	628
39	2014	Stoppe M, Thomä E, Liebert UG, et al.	49	m	Psoriasis	Autoimmune disease	Alive	1156
40	2016	Berntsson SG, Katsarogiannis E, Lourenço F, Moraes-Fontes MF.	34	f	SLE	Autoimmune disease	Deceased	365

© 2024 Möhn N et al. *JAMA Neurology.*

41	2015	Nieuwkamp DJ, Murk JL, van Oosten BW, et al.	64	f	Psoriasis	Autoimmune disease	Deceased	53
42	2017	Agarwal S, Patrick J, Jones J, Smith R, Coles A, Jayne D.	33	f	Behçet's disease	Autoimmune disease	Deceased	74
43	2018	Scabini S, Trunfio M, Pirriatore V, et al.	50	f	Mixed connective-tissue disease, Sjögren syndrome	Autoimmune disease	Deceased	60
44	2017	Scholten P, Kralt P, Jacobs B.	57	m	Sarcoidosis	Autoimmune disease	Deceased	60
45	2019	Law LY, Tan I, Prowse M, Sean Riminton D, Reddel SW.	64	f	SLE	Autoimmune disease	Deceased	540
46	2021	Onwubiko IN, Taneja K, Gupta N, Mukherjee A.	65	f	Sjögren syndrome	Autoimmune disease	Deceased	8
47	2020	Elbadri M, Plant G.	49	m	Sarcoidosis	Autoimmune disease	Deceased	56
48	2020	Guduru M, Bendi VS, Bebawy MS, Bande D, Matta A.	79	f	Rheumatoid arthritis	Autoimmune disease	Deceased	30
49	2017	Kmezic I, Weinberg J, Hauzenberger D, et al.	81	m	Polycythemia vera	Autoimmune disease	Deceased	60
50	2016	de Regt MJ, Murk JL, Schneider-Hohendorf T, Wattjes MP, Hoepelman AI, Arends JE.	75	f	Rheumatoid arthritis	Autoimmune disease	Deceased	35
51	2019	Zhong M, Kempster PA, Phan TG.	60	f	SLE	Autoimmune disease	Deceased	90
52	2017	Gocmen R, Acar NP, Cagdas D, Kurne A.	23	m	Hyperimmunoglobulin IgE syndrome, DLBCL	Autoimmune disease	Deceased	210
53	2018	Ueno T, Sato N, Kon T, et al.	47	f	Myasthenia gravis, invasive thymoma	Autoimmune disease	Deceased	46
54	2018	Aghoram R, Narayan SK.	36	m	Idiopathic CD4+ lymphocytopenia	Lymphopenia	Deceased	120
55	2018	Kano Y, Inoue H, Sakurai K, et al.	75	m	Idiopathic CD4+ lymphocytopenia	Lymphopenia	Deceased	870
56	2016	Aotsuka Y, Uzawa A, Nishimura K, et al.	66	m	Idiopathic CD4+ lymphocytopenia	Lymphopenia	Deceased	21
57	2019	Aggarwal D, Tom JP, Chatterjee D, Goyal M.	45	m	Idiopathic CD4+ lymphocytopenia	Lymphopenia	Deceased	14
58	2018	Hautala TJ, Perelygina L, Vuorinen T, et al.	29	f	Combined primary immunodeficiency	Lymphopenia	Deceased	185
59	2020	Sutton P, Raslau F, Sudhakar P.	87	m	Idiopathic CD4+ lymphocytopenia	Lymphopenia	Deceased	15
60	2016	Kromm JA, Power C, Blevins G, Larratt L, van Landeghem FK, Rempel J.	70	m	CLL	Lymphoproliferative	Deceased	60
61	2015	Kreps EO, Van Hoof A, Reyniers R.	65	m	CLL	Lymphoproliferative	Deceased	180
62	2018	Ramadhani P, Bramantono B, Sedana MP.	67	f	CLL	Lymphoproliferative	Deceased	90

© 2024 Möhn N et al. *JAMA Neurology.*

63	2018	Ikegawa S, Fujii N, Tadokoro K, et al.	52	m	DLBCL	Lymphoproliferative	Deceased	138
64	2017	Lutz M, Schulze AB, Rebber E, et al.	65	m	CLL	Lymphoproliferative	Deceased	49
65	2020	Vogrig A, Gigli GL, Nilo A, et al.	64	f	CLL	Lymphoproliferative	Deceased	360
66	2019	Anderson S, Kiernan M, Ho PJ.	60	f	Multiple myeloma	Lymphoproliferative	Deceased	120
67	2020	Bennett KM, Storrar N, Johnson P, Fernandes PM.	67	m	Multiple myeloma	Lymphoproliferative	Deceased	14
68	2015	Sano Y, Nakano Y, Omoto M, et al.	66	m	NHL	Lymphoproliferative	Deceased	300
69	2018	Yamashita Y, Kusakabe S, Toda J, et al.	37	m	T-lympho-blastic lymphoma	Lymphoproliferative	Deceased	84
70	2019	Muto R, Sugita Y, Momosaki S, Ito Y, Wakugawa Y, Ohshima K.	63	f	NHL	Lymphoproliferative	Deceased	365
71	2021	Mian A, Andrapalliyal N, Weathers AL, Pohlman B, Hill BT.	61	f	LBCL (germinal center B-cell subtype)	Lymphoproliferative	Deceased	13
72	2021	Sanjo N, Nose Y, Miyamoto S, et al.	70	f	Adult T-cell leukemia	Lymphoproliferative	Deceased	300
73	2017	Brigo F, Pagani E, Tezzon F, Masi E, Nardone R.	60	m	Multiple myeloma	Lymphoproliferative	Deceased	75
74	2020	Forryan J, Yong J.	68	m	CLL	Lymphoproliferative	Deceased	150
75	2020	Trociukas I, Zirnis AE, Beļajeva L, Rivkina A, Lejniece S.	53	f	Follicular lymphoma	Lymphoproliferative	Deceased	60
76	2019	Yeung J, van Hal S, Ho PJ.	62	m	Multiple myeloma	Lymphoproliferative	Deceased	42
77	2020	Lucijanic M, Jaksic O.	47	f	Follicular lymphoma	Lymphoproliferative	Deceased	120
78	2020	Knight K, Chien S, Koutsavlis I, Campbell V.	59	m	Multiple myeloma	Lymphoproliferative	Deceased	14
79	2018	Yuan C, Deberardinis C, Patel R, et al.	59	f	AML, autoimmune hemolytic anemia	Mixed condition	Deceased	60
80	2018	Windpessl M, Burgstaller S, Kronbichler A, et al.	45	m	Kidney transplant, PTLD	Mixed condition	Deceased	21
81	2014	Lee HC, Mulanovich V, Nieto Y.	62	m	AML	Lymphoproliferative	Deceased	46
82	2014	Lee HC, Mulanovich V, Nieto Y.	69	m	AML	Lymphoproliferative	Deceased	49
83	2014	Isidoro L, Pires P, Rito L, Cordeiro G.	85	f	CLL	Lymphoproliferative	Deceased	305
84	2014	Di Pauli F, Berger T, Walder A, et al.	45	f	CLL	Lymphoproliferative	Deceased	107

© 2024 Möhn N et al. *JAMA Neurology.*

85	2014	Kaufman GP, Aksamit AJ, Klein CJ, Yi ES, Delone DR, Litzow MR.	67	m	ALL	Lymphoproliferative	Deceased	30
86	2014	Lach B, Connolly B, Wüthrich C, Koralnik IJ.	65	m	Rheumatoid arthritis	Autoimmune disease	Deceased	61
87	2014	Fredericks CA, Kvam KA, Bear J, Crabtree GS, Josephson SA.	48	f	SLE	Autoimmune disease	Deceased	30

eTable 3: Detailed characteristics of best supportive treatment-controls published within the literature

Characteristics of patients published within the literature used for historical BMT-control group. f = female; m = male; SLE = systemic lupus erythematosus; NHL = Non-Hodgkin lymphoma; CLL = Chronic lymphocytic leukemia; AML = Acute myeloid leukemia; DLBCL = Diffuse large B-cell lymphoma; ALL = Acute lymphatic leukemia

© 2024 Möhn N et al. *JAMA Neurology.*

eREFERENCES

1. Hopfner F, Mohn N, Eiz-Vesper B, et al. Allogeneic BK Virus-Specific T-Cell Treatment in 2 Patients With Progressive Multifocal Leukoencephalopathy. *Neurol Neuroimmunol Neuroinflamm*. 2021;8(4):10.1212/NXI.0000000000001020. Print 2021 Jul. doi: e1020 [pii].

2. Bonifacius A, Lamottke B, Tischer-Zimmermann S, et al. Patient-tailored adoptive immunotherapy with EBV-specific T cells from related and unrelated donors. *The Journal of clinical investigation*. 2023;133(12):1-14. doi: 10.1172/JCI163548.

3. IARC Working Group on the Evaluation of Carcinogenic Risks to Humans. Malaria and some polyomaviruses (SV40, BK, JC, and Markel cell viruses). *IARC Monogr Eval Carcinog Risks Hum*. 2014;104:9-350.

4. Schulze Lammers FC, Bonifacius A, Tischer-Zimmermann S, et al. Antiviral T-Cell Frequencies in a Healthy Population: Reference Values for Evaluating Antiviral Immune Cell Profiles in Immunocompromised Patients. *J Clin Immunol*. 2022;42(3):546-558. doi: 10.1007/s10875-021-01205-1.

5. Tischer S, Dieks D, Sukdolak C, et al. Evaluation of suitable target antigens and immunoassays for high-accuracy immune monitoring of cytomegalovirus and Epstein-Barr virus-specific T cells as targets of interest in immunotherapeutic approaches. *J Immunol Methods*. 2014;408:101-113. doi: 10.1016/j.jim.2014.05.011.

6. Priesner C, Esser R, Tischer S, et al. Comparative Analysis of Clinical-Scale IFN-γ-Positive T-Cell Enrichment Using Partially and Fully Integrated Platforms. *Front Immunol*. 2016;7:393. doi: 10.3389/fimmu.2016.00393.

7. Bonifacius A, Tischer-Zimmermann S, Santamorena MM, et al. Rapid Manufacturing of Highly Cytotoxic Clinical-Grade SARS-CoV-2-specific T Cell Products Covering SARS-CoV-2 and Its Variants for Adoptive T Cell Therapy. *Front Bioeng Biotechnol*. 2022;10:867042. doi: 10.3389/fbioe.2022.867042.

8. Tischer S, Priesner C, Heuft H, et al. Rapid generation of clinical-grade antiviral T cells: selection of suitable T-cell donors and GMP-compliant manufacturing of antiviral T cells. *J Transl Med*. 2014;12:336. doi: 10.1186/s12967-014-0336-5.

9. Lee HC, Mulanovich V, Nieto Y. Progressive multifocal leukoencephalopathy after allogeneic bone marrow transplantation for acute myeloid leukemia. *J Natl Compr Canc Netw*. 2014;12(12):1660-1664; quiz 1664. doi: 10.6004/jnccn.2014.0167.

10. Fredericks CA, Kvam KA, Bear J, Crabtree GS, Josephson SA. A case of progressive multifocal leukoencephalopathy in a lupus patient treated with belimumab. *Lupus*. 2014;23(7):711-713. doi: 10.1177/0961203314524292.

11. Lach B, Connolly B, Wüthrich C, Koralnik IJ. Inflammatory infratentorial progressive multifocal leukoencephalopathy in a patient with rheumatoid arthritis. *Neuropathology*. 2014;34(1):39-44. doi: 10.1111/neup.12045.

12. Kaufman GP, Aksamit AJ, Klein CJ, Yi ES, Delone DR, Litzow MR. Progressive multifocal leukoencephalopathy: a rare infectious complication following allogeneic hematopoietic cell transplantation (HCT). *Eur J Haematol*. 2014;92(1):83-87. doi: 10.1111/ejh.12208.

13. Di Pauli F, Berger T, Walder A, et al. Progressive multifocal leukoencephalopathy complicating untreated chronic lymphatic leukemia: case report and review of the literature. *J Clin Virol*. 2014;60(4):424-427. doi: 10.1016/j.jcv.2014.05.007.

14. Isidoro L, Pires P, Rito L, Cordeiro G. Progressive multifocal leukoencephalopathy in a patient with chronic lymphocytic leukaemia treated with alemtuzumab. *BMJ Case Rep*. 2014;2014:bcr2013201781. doi: 10.1136/bcr-2013-201781.

© 2024 Möhn N et al. *JAMA Neurology.*

15. Lee HC, Mulanovich V, Nieto Y. Progressive multifocal leukoencephalopathy after allogeneic bone marrow transplantation for acute myeloid leukemia. *J Natl Compr Canc Netw*. 2014;12(12):1660-1664; quiz 1664. doi: 10.6004/jnccn.2014.0167.

16. Windpessl M, Burgstaller S, Kronbichler A, et al. Progressive Multifocal Leukoencephalopathy Following Combined Rituximab-Based Immune-Chemotherapy for Post-transplant Lymphoproliferative Disorder in a Renal Transplant Recipient: A Case Report. *Transplant Proc*. 2018;50(3):881-883. doi: 10.1016/j.transproceed.2018.01.004.

17. Yuan C, Deberardinis C, Patel R, et al. Progressive multifocal leukoencephalopathy after allogeneic stem cell transplantation: Case report and review of the literature. *Transpl Infect Dis*. 2018;20(3):e12879. doi: 10.1111/tid.12879.

18. Knight K, Chien S, Koutsavlis I, Campbell V. Progressive multifocal leukoencephalopathy following five lines of therapy and three autologous bone marrow transplants for multiple myeloma. *BMJ Case Rep*. 2020;13(3):e233552. doi: 10.1136/bcr-2019-233552.

19. Lucijanic M, Jaksic O. Progressive multifocal leukoencephalopathy during rituximab maintenance after rituximab and bendamustine treatment for relapsed follicular lymphoma. *Ann Hematol*. 2020;99(9):2191-2192. doi: 10.1007/s00277-020-04143-x.

20. Yeung J, van Hal S, Ho PJ. Prolonged Immunosuppression in Relapsed, Refractory Multiple Myeloma Leading to Cerebral Toxoplasmosis and Progressive Multifocal Leukoencephalopathy. *Clin Lymphoma Myeloma Leuk*. 2019;19(12):e625-e628. doi: 10.1016/j.clml.2019.09.617.

21. Trociukas I, Zirnis AE, Beļajeva L, Rivkina A, Lejniece S. Progressive multifocal encephalopathy in a patient with non-Hodgkin follicular lymphoma. *Exp Oncol*. 2020;42(3):238-241. doi: 10.32471/exp-oncology.2312-8852.vol-42-no-3.15198.

22. Forryan J, Yong J. Rapid cognitive decline in a patient with chronic lymphocytic leukaemia: a case report. *J Med Case Rep*. 2020;14(1):39. doi: 10.1186/s13256-020-2360-9.

23. Brigo F, Pagani E, Tezzon F, Masi E, Nardone R. Lenalidomide-associated progressive multifocal leukoencephalopathy. *Leuk Lymphoma*. 2017;58(10):2514-2515. doi: 10.1080/10428194.2017.1292355.

24. Sanjo N, Nose Y, Miyamoto S, et al. Early Pathological JC Virus Lesions in a Patient without Any MRI-based Indications. *Intern Med*. 2021;60(8):1279-1282. doi: 10.2169/internalmedicine.6040-20.

25. Mian A, Andrapalliyal N, Weathers AL, Pohlman B, Hill BT. Late occurrence of progressive multifocal leukoencephalopathy after anti-CD19 chimeric antigen receptor T-cell therapy. *Eur J Haematol*. 2021;106(4):584-588. doi: 10.1111/ejh.13583.

26. Muto R, Sugita Y, Momosaki S, Ito Y, Wakugawa Y, Ohshima K. An autopsy case of progressive multifocal leukoencephalopathy after rituximab therapy for malignant lymphoma. *Neuropathology*. 2019;39(1):58-63. doi: 10.1111/neup.12526.

27. Yamashita Y, Kusakabe S, Toda J, et al. Sequential Onset of Varicella-Zoster Virus Encephalomeningitis and Progressive Multifocal Leukoencephalopathy in an Allogeneic Hematopoietic Stem Cell Transplant Recipient. *Exp Clin Transplant*. 2018;16(5):628-630. doi: 10.6002/ect.2016.0077.

28. Sano Y, Nakano Y, Omoto M, et al. Rituximab-associated progressive multifocal leukoencephalopathy derived from non-Hodgkin lymphoma: neuropathological findings and results of mefloquine treatment. *Intern Med*. 2015;54(8):965-970. doi: 10.2169/internalmedicine.54.2308.

© 2024 Möhn N et al. *JAMA Neurology.*

29. Bennett KM, Storrar N, Johnson P, Fernandes PM. Progressive multifocal leukoencephalopathy (PML) following autologous peripheral blood stem cell transplantation for multiple myeloma. *Clin Case Rep*. 2020;8(6):938-943. doi: 10.1002/ccr3.2612.

30. Anderson S, Kiernan M, Ho PJ. Lenalidomide-related Progressive Multifocal Leukoencephalopathy: A Case Report and Review of Drug-related Cases in Multiple Myeloma. *Clin Lymphoma Myeloma Leuk*. 2019;19(4):e169-e171. doi: 10.1016/j.clml.2018.12.021.

31. Vogrig A, Gigli GL, Nilo A, et al. Epilepsia partialis continua revealing idelalisib-associated PML-IRIS: clinical and pathological features. *J Neurovirol*. 2020;26(3):437-441. doi: 10.1007/s13365-019-00821-3.

32. Lutz M, Schulze AB, Rebber E, et al. Progressive Multifocal Leukoencephalopathy after Ibrutinib Therapy for Chronic Lymphocytic Leukemia. *Cancer Res Treat*. 2017;49(2):548-552. doi: 10.4143/crt.2016.110.

33. Ikegawa S, Fujii N, Tadokoro K, et al. Progressive multifocal leukoencephalopathy after T-cell replete HLA-haploidentical transplantation with post-transplantation cyclophosphamide graft-versus-host disease prophylaxis. *Transpl Infect Dis*. 2018;20(2):e12850. doi: 10.1111/tid.12850.

34. Ramadhani P, Bramantono B, Sedana MP. A Chronic Lymphocytic Leukemia Patient with Progressive Multifocal Leukoencephalopathy Caused by John Cunningham Virus. *Acta Med Indones*. 2018;50(2):151-158.

35. Kreps EO, Van Hoof A, Reyniers R. Subacute visual loss as the sole presenting feature in progressive multifocal leukoencephalopathy. *Clin Exp Ophthalmol*. 2015;43(6):599-600. doi: 10.1111/ceo.12504.

36. Kromm JA, Power C, Blevins G, Larratt L, van Landeghem FKH, Rempel J. Rapid Multifocal Neurologic Decline in an Immunocompromised Patient. *JAMA Neurol*. 2016;73(2):226-231. doi: 10.1001/jamaneurol.2015.2658.

37. Sutton P, Raslau F, Sudhakar P. A Rare Case of Cerebellar Progressive Multifocal Leukoencephalopathy due to Idiopathic CD4 Lymphocytopenia. *Mult Scler Relat Disord*. 2020;46:102531. doi: 10.1016/j.msard.2020.102531.

38. Hautala TJ, Perelygina L, Vuorinen T, et al. Nitazoxanide May Modify the Course of Progressive Multifocal Leukoencephalopathy. *J Clin Immunol*. 2018;38(1):4-6. doi: 10.1007/s10875-017-0463-x.

39. Aggarwal D, Tom JP, Chatterjee D, Goyal M. Progressive multifocal leukoencephalopathy in idiopathic CD4+ lymphocytopenia: A case report and review of literature. *Neuropathology*. 2019;39(6):467-473. doi: 10.1111/neup.12599.

40. Aotsuka Y, Uzawa A, Nishimura K, et al. Progressive Multifocal Leukoencephalopathy Localized in the Cerebellum and Brainstem Associated with Idiopathic CD4(+) T Lymphocytopenia. *Intern Med*. 2016;55(12):1645-1647. doi: 10.2169/internalmedicine.55.6649.

41. Kano Y, Inoue H, Sakurai K, et al. [Idiopathic CD4-positive lymphocytopenia-associated progressive multifocal leukoencephalopathy confirmed by brain biopsy following negative results of repeated CSF-JC-virus tests: a case report]. *Rinsho Shinkeigaku*. 2018;58(12):750-755. doi: 10.5692/clinicalneurol.cn-001227.

42. Aghoram R, Narayan SK. Progressive multifocal leukoencephalopathy in idiopathic CD4+ lymphocytopenia. *J Neurovirol*. 2018;24(4):526-528. doi: 10.1007/s13365-018-0638-0.

43. Ueno T, Sato N, Kon T, et al. Progressive multifocal leukoencephalopathy associated with thymoma with immunodeficiency: a case report and literature review. *BMC Neurol*. 2018;18(1):37. doi: 10.1186/s12883-018-1041-4.

© 2024 Möhn N et al. *JAMA Neurology.*

44. Gocmen R, Acar NP, Cagdas D, Kurne A. Progressive multifocal leukoencephalopathy in a patient with lymphoma and presumptive hyper IgE syndrome. *J Neurovirol*. 2017;23(4):632-636. doi: 10.1007/s13365-017-0536-x.

45. Zhong M, Kempster PA, Phan TG. John Cunningham virus granule cell neuronopathy in a mildly immunosuppressed patient with systemic lupus erythematosus. *Intern Med J*. 2019;49(6):804-805. doi: 10.1111/imj.14318.

46. de Regt MJA, Murk J, Schneider-Hohendorf T, Wattjes MP, Hoepelman AIM, Arends JE. Progressive multifocal leukoencephalopathy and black fungus in a patient with rheumatoid arthritis without severe lymphocytopenia. *JMM Case Rep*. 2016;3(4):e005053. doi: 10.1099/jmmcr.0.005053.

47. Kmezic I, Weinberg J, Hauzenberger D, et al. An unusual cause of fatal rapid-onset ataxia plus syndrome. *Cerebellum Ataxias*. 2017;4:5. doi: 10.1186/s40673-017-0063-9.

48. Guduru M, Bendi VS, Bebawy MS, Bande D, Matta A. Posterior Fossa Progressive Multifocal Leukoencephalopathy Secondary to Rituximab. *Cureus*. 2020;12(10):e10888. doi: 10.7759/cureus.10888.

49. Elbadri M, Plant G. Progressive multifocal leukoencephalopathy as the first presentation of sarcoidosis. *BMJ Case Rep*. 2020;13(8):e232636. doi: 10.1136/bcr-2019-232636.

50. Onwubiko IN, Taneja K, Gupta N, Mukherjee A. Unusual Case of Progressive Multifocal Leukoencephalopathy in a Patient With Sjögren Syndrome. *Am J Forensic Med Pathol*. 2021;42(2):186-190. doi: 10.1097/PAF.0000000000000656.

51. Law LY, Tan I, Prowse M, Sean Riminton D, Reddel SW. Progressive multifocal leukoencephalopathy in a patient with systemic lupus erythematosus: Clues to early diagnosis. *J Clin Neurosci*. 2019;67:261-263. doi: 10.1016/j.jocn.2019.06.030.

52. Scholten P, Kralt P, Jacobs B. Posterior fossa progressive multifocal leukoencephalopathy: first presentation of an unknown autoimmune disease. *BMJ Case Rep*. 2017;2017:bcr2017220990, bcr-220990. doi: 10.1136/bcr-2017-220990.

53. Scabini S, Trunfio M, Pirriatore V, et al. Antiviral activity of maraviroc plus mirtazapine in a low-risk HIV-negative patient with progressive multifocal leukoencephalopathy. *Infez Med*. 2018;26(2):160-163.

54. Agarwal S, Patrick J, Jones J, Smith R, Coles A, Jayne D. Progressive multifocal leucoencephalopathy with Behçet's disease: an insight into pathophysiology. *Rheumatology (Oxford)*. 2017;56(4):668-670. doi: 10.1093/rheumatology/kew404.

55. Nieuwkamp DJ, Murk J, van Oosten BW, et al. PML in a patient without severe lymphocytopenia receiving dimethyl fumarate. *N Engl J Med*. 2015;372(15):1474-1476. doi: 10.1056/NEJMc1413724.

56. Berntsson SG, Katsarogiannis E, Lourenço F, Moraes-Fontes MF. Progressive Multifocal Leukoencephalopathy and Systemic Lupus Erythematosus: Focus on Etiology. *Case Rep Neurol*. 2016;8(1):59-65. doi: 10.1159/000444874.

57. Stoppe M, Thomä E, Liebert UG, et al. Cerebellar manifestation of PML under fumarate and after efalizumab treatment of psoriasis. *J Neurol*. 2014;261(5):1021-1024. doi: 10.1007/s00415-014-7311-1.

58. Yoshida H, Ohshima K, Toda J, et al. Significant improvement following combination treatment with mefloquine and mirtazapine in a patient with progressive multifocal leukoencephalopathy after allogeneic peripheral blood stem cell transplantation. *Int J Hematol*. 2014;99(1):95-99. doi: 10.1007/s12185-013-1471-0.

© 2024 Möhn N et al. *JAMA Neurology.*

59. Mungunkhuyag M, Harada M, Abe T, Fujita K, Matsui N, Kaji R. Longitudinal monitoring with multiple MR techniques in a case of progressive multifocal leukoencephalopathy associated with multiple myeloma. *Magn Reson Med Sci*. 2014;13(1):55-59. doi: 10.2463/mrms.2013-0037.

60. Berger MD, Meisel A, Andres M, Schanz U, Schwarz U, Stussi G. Unusual case of progressive multifocal leukoencephalopathy after allogeneic hematopoietic stem-cell transplantation. *J Clin Oncol*. 2014;32(9):33. doi: 10.1200/JCO.2012.47.5194.

61. Alstadhaug KB, Croughs T, Henriksen S, et al. Treatment of progressive multifocal leukoencephalopathy with interleukin 7. *JAMA Neurol*. 2014;71(8):1030-1035. doi: 10.1001/jamaneurol.2014.825.

62. Skovrlj B, Rasouli J, Caridi J, Taylor WM, Galyon DD. Progressive multifocal leukoencephalopathy presenting as a single ring-enhancing lesion. *Clin Neurol Neurosurg*. 2014;122:77-79. doi: 10.1016/j.clineuro.2014.04.019.

63. Godbole MM, Barr PM. Complete recovery of late onset progressive multifocal leukoencephalopathy related to treatment with chemoimmunotherapy: A case report. *Leuk Res*. 2020;90:106309. doi: 10.1016/j.leukres.2020.106309.

64. Peña M, Presas-Rodríguez S, Ribera J. Efficacy of mefloquine and mirtazapine on progressive multifocal leukoencephalopathy in a patient with peripheral T-cell lymphoma. *Med Clin (Barc)*. 2019;153(9):e47-e48. doi: 10.1016/j.medcli.2019.01.004.

65. Amano E, Ozaki K, Ishibashi S, Sanjo N, Yokota T. Remarkable improvement in progressive multifocal leukoencephalopathy following acute pyelonephritis with bacteremia. *J Clin Neurosci*. 2019;62:226-228. doi: 10.1016/j.jocn.2018.12.034.

66. Kelly D, Monaghan B, McMahon E, et al. Progressive multifocal leukoencephalopathy secondary to rituximab-induced immunosuppression and the presence of John Cunningham virus: a case report and literature review. *Radiol Case Rep*. 2016;11(3):251-254. doi: 10.1016/j.radcr.2016.06.003.

67. Martinot M, Ahle G, Petrosyan I, et al. Progressive Multifocal Leukoencephalopathy after Treatment with Nivolumab. *Emerg Infect Dis*. 2018;24(8):1594-1596. doi: 10.3201/eid2408.180460.

68. Mosna K, Ladicka M, Drgona L, et al. Ibrutinib treatment of mantle cell lymphoma complicated by progressive multifocal leukoencephalopathy. *Int J Clin Pharmacol Ther*. 2020;58(6):343-350. doi: 10.5414/CP203663.

69. Nakamura R, Kitamura A, Tsukamoto T, et al. The Effect of Intravenous Methylprednisolone on Recurrent Exacerbation in Hematologic Malignancy-associated Progressive Multifocal Leukoencephalopathy. *Intern Med*. 2021;60(8):1287-1291. doi: 10.2169/internalmedicine.5917-20.

70. Hsiehchen D, Arasaratnam R, Raj K, Froehlich T, Anderson L. Ibrutinib Use Complicated by Progressive Multifocal Leukoencephalopathy. *Oncology*. 2018;95(5):319-322. doi: 10.1159/000490617.

71. Pasca M, Picchioni A, Mazzeo S, et al. A case of recurrent progressive multifocal leukoencephalopathy after human stem cell transplant, with detection of John Cunningham virus and human herpesvirus 6 on cerebrospinal fluid, treated with Mirtazapine, Olanzapine and Foscarnet. *Intractable Rare Dis Res*. 2019;8(4):275-278. doi: 10.5582/irdr.2019.01107.

72. Lam W, Al-Shaibani Z, Kumar D, et al. Progressive multifocal leukoencephalopathy due to John Cunningham (JC) virus following allogeneic haematopoietic cell transplantation. *Antivir Ther*. 2017;22(8):721-725. doi: 10.3851/IMP3162.

73. Sanjo N, Kina S, Shishido-Hara Y, et al. Progressive Multifocal Leukoencephalopathy with Balanced CD4/CD8 T-Cell Infiltration and Good Response to Mefloquine Treatment. *Intern Med*. 2016;55(12):1631-1635. doi: 10.2169/internalmedicine.55.6051.
© 2024 Möhn N et al. *JAMA Neurology.*

74. Garrote H, de la Fuente A, Oña R, et al. Long-term survival in a patient with progressive multifocal leukoencephalopathy after therapy with rituximab, fludarabine and cyclophosphamide for chronic lymphocytic leukemia. *Exp Hematol Oncol*. 2015;4:8. doi: 10.1186/s40164-015-0003-4.

75. Yokokawa K, Hisahara S, Matsuura Y, et al. Progressive multifocal leukoencephalopathy after autologous peripheral blood stem cell transplantation in a patient with multiple myeloma treated with combination therapy. *J Neurol Sci*. 2016;368:304-306. doi: 10.1016/j.jns.2016.07.045.

76. Ueno H, Kikumto M, Takebayashi Y, et al. Pomalidomide-associated progressive multifocal leukoencephalopathy in multiple myeloma: cortical susceptibility-weighted imaging hypointense findings prior to clinical deterioration. *J Neurovirol*. 2020;26(3):452-455. doi: 10.1007/s13365-020-00845-0.

77. Masuoka K, Akagawa Y, Hanashiro R, Yamaguchi M, Oota H, Kiyozuka T. [Progressive multifocal leukoencephalopathy developing subsequent to cord blood transplantation in a patient with severe aplastic anemia]. *Rinsho Ketsueki*. 2019;60(2):93-98. doi: 10.11406/rinketsu.60.93.

78. Castro-Sánchez MV, Villagrán-García M, Romero-Imbroda J. Acute disseminated encephalomyelitis due to JC virus infection as the onset of non-Hodgkin's lymphoma. *Med Clin (Barc)*. 2019;152(9):e49-e50. doi: 10.1016/j.medcli.2018.07.021.

79. Oza A, Rettig MP, Powell P, et al. Interleukin-15 superagonist (N-803) treatment of PML and JCV in a post-allogeneic hematopoietic stem cell transplant patient. *Blood Adv*. 2020;4(11):2387-2391. doi: 10.1182/bloodadvances.2019000664.

80. Gupta HV, Gokden M, Ramakrishnaiah RH, Archer RL. Progressive multifocal leukoencephalopathy mimicking glioma in a patient with idiopathic CD4 lymphocytopenia. *Acta Neurol Belg*. 2016;116(3):391-394. doi: 10.1007/s13760-015-0532-y.

81. Harel A, Horng S, Gustafson T, Ramineni A, Farber RS, Fabian M. Successful treatment of progressive multifocal leukoencephalopathy with recombinant interleukin-7 and maraviroc in a patient with idiopathic CD4 lymphocytopenia. *J Neurovirol*. 2018;24(5):652-655. doi: 10.1007/s13365-018-0657-x.

82. Dato C, Elefante A, Coppola C, et al. "Borderline" idiopathic CD4+ T-cell lymphocytopenia presenting with atypical progressive multifocal leukoencephalopathy. *J Neuroimmunol*. 2020;349:577420. doi: 10.1016/j.jneuroim.2020.577420.

83. Nambirajan A, Suri V, Kataria V, Sharma MC, Goyal V. Progressive multifocal leukoencephalopathy in a 44-year old male with idiopathic CD4+ T-lymphocytopenia treated with mirtazapine and mefloquine. *Neurol India*. 2017;65(5):1061-1064. doi: 10.4103/neuroindia.NI_535_16.

84. Kurmann R, Weisstanner C, Kardas P, et al. Progressive multifocal leukoencephalopathy in common variable immunodeficiency: mitigated course under mirtazapine and mefloquine. *J Neurovirol*. 2015;21(6):694-701. doi: 10.1007/s13365-015-0340-4.

85. Tirelli L, Rosini F, Rufa A, et al. Clinical and MRI improvement in a case of progressive multifocal leukoencephalopathy. *Neurol Sci*. 2017;38(8):1517-1519. doi: 10.1007/s10072-017-2914-9.

86. Nosaki Y, Ohyama K, Watanabe M, et al. Simultaneous Development of Progressive Multifocal Leukoencephalopathy and Cryptococcal Meningitis during Methotrexate and Infliximab Treatment. *Intern Med*. 2019;58(18):2703-2709. doi: 10.2169/internalmedicine.2570-18.

87. Ellrichmann G, Behrendt V, Grunwald C, Schlottmann R, Lukas C, Gold R. Cidofovir for treatment of progressive multifocal leukoencephalopathy in a newly diagnosed sarcoidosis patient - a valid therapeutic option?. *J Neurol Sci*. 2016;362:341-343. doi: 10.1016/j.jns.2016.02.011.

© 2024 Möhn N et al. *JAMA Neurology.*

88. Bartsch T, Rempe T, Wrede A, et al. Progressive neurologic dysfunction in a psoriasis patient treated with dimethyl fumarate. *Ann Neurol*. 2015;78(4):501-514. doi: 10.1002/ana.24471.

89. Dammeier N, Schubert V, Hauser T, Bornemann A, Bischof F. Case report of a patient with progressive multifocal leukoencephalopathy under treatment with dimethyl fumarate. *BMC Neurol*. 2015;15:108. doi: 10.1186/s12883-015-0363-8.

90. Nishigori R, Warabi Y, Shishido-Hara Y, et al. Inflammatory Cerebellar PML with a CD4/CD8 Ratio of 2.9 Showed a Favorable Prognosis in a Patient with Rheumatoid Arthritis. *Intern Med*. 2019;58(22):3323-3329. doi: 10.2169/internalmedicine.3038-19.

91. Trentalange A, Calcagno A, Ghisetti V, et al. Clearance of cerebrospinal fluid JCV DNA with mirtazapine in a patient with progressive multifocal leukoencephalopathy and sarcoidosis. *Antivir Ther*. 2016;21(7):633-635. doi: 10.3851/IMP3032.

92. Cheng C, Su J, Chen Y, Lin Y, Huang Y, Li K. Progressive multifocal leukoencephalopathy in a 27-year-old lady with systemic lupus erythematosus - rheumatoid arthritis overlap syndrome. *J Formos Med Assoc*. 2019;118(11):1560-1565. doi: 10.1016/j.jfma.2019.05.021.

93. Ishikawa Y, Kasuya T, Ishikawa J, Fujiwara M, Kita Y. A case of developing progressive multifocal leukoencephalopathy while using rituximab and mycophenolate mofetil in refractory systemic lupus erythematosus. *Ther Clin Risk Manag*. 2018;14:1149-1153. doi: 10.2147/TCRM.S167109.

94. Ikeda J, Matsushima A, Ishii W, et al. Brain Biopsy Is More Reliable than the DNA test for JC Virus in Cerebrospinal Fluid for the Diagnosis of Progressive Multifocal Leukoencephalopathy. *Intern Med*. 2017;56(10):1231-1234. doi: 10.2169/internalmedicine.56.7689.

95. Ishii J, Shishido-Hara Y, Kawamoto M, et al. A Punctate Magnetic Resonance Imaging Pattern in a Patient with Systemic Lupus Erythematosus Is an Early Sign of Progressive Multifocal Leukoencephalopathy: A Clinicopathological Study. *Intern Med*. 2018;57(18):2727-2734. doi: 10.2169/internalmedicine.0696-17.

96. Boumaza X, Bonneau B, Roos-Weil D, et al. Progressive Multifocal Leukoencephalopathy Treated by Immune Checkpoint Inhibitors. *Ann Neurol*. 2023;93(2):257-270. doi: 10.1002/ana.26512.

97. Heckler I, Venkataraman I. Phosphorylated neurofilament heavy chain: a potential diagnostic biomarker in amyotrophic lateral sclerosis. *J Neurophysiol*. 2022;127(3):737-745. doi: 10.1152/jn.00398.2021.

98. Teunissen CE, Dijkstra C, Polman C. Biological markers in CSF and blood for axonal degeneration in multiple sclerosis. *The Lancet Neurology*. 2005;4(1):32-41. doi: 10.1016/S1474-4422(04)00964-0.

99. SantaCruz KS, Roy G, Spigel J, Bearer EL. Neuropathology of JC virus infection in progressive multifocal leukoencephalopathy in remission. *World J Virol*. 2016;5(1):31-37. doi: 10.5501/wjv.v5.i1.31.

© 2024 Möhn N et al. *JAMA Neurology.*
