# Supplementary material for: Directly Isolated Allogeneic Virus–Specific T Cells in Progressive Multifocal Leukoencephalopathy
Source: JAMA Neurol. 2024 Oct 7;81(11):1187–98. doi: 10.1001/jamaneurol.2024.3324 (PMC11459361; doi:10.1001/jamaneurol.2024.3324)
Supplement: Supplement 2. — Data Sharing Statement. [file jamaneurol-e243324-s002.pdf]

Data Sharing Statement

Möhn. Directly Isolated Allogeneic Virus–Specific T Cells in Progressive Multifocal Leukoencephalopathy. *JAMA Neurol*. Published October 07, 2024. doi:10.1001/jamaneurol.2024.3324

Data
Data available: No

Additional Information
Explanation for why data not available: The manuscript presents detailed information on individual patients in an anonymized form. To protect privacy in the context of the compassionate use treatment, no further detailed patient data is provided. We can provide the data on request.
